# Flexible n‑Channel Organic Transistors with Low Contact Resistance

**DOI:** 10.1021/acsami.5c17364

**Published:** 2025-12-22

**Authors:** Sabrina Steffens, Tobias Wollandt, Karla Cordero-Solano, Robert Eichelmann, Alexander Kochan, Xiuming Sun, Florian Letzkus, Joachim N. Burghartz, Sabine Ludwigs, Lutz H. Gade, Hagen Klauk

**Affiliations:** a 28326 Max Planck Institute for Solid State Research, Heisenbergstraße 1, Stuttgart 70569, Germany; b Anorganisch-Chemisches Institut, 9144 Universität Heidelberg, Im Neuenheimer Feld 270, Heidelberg 69120, Germany; c IPOC - Functional Polymers, Institute of Polymer Chemistry (IPOC), 9149 Universität Stuttgart, Pfaffenwaldring 55, Stuttgart 70569, Germany; d 529944 Institut für Mikroelektronik Stuttgart (IMS CHIPS), Allmandring 30a, Stuttgart 70569, Germany

**Keywords:** organic thin-film transistors, n-channel organic transistors, small-molecule organic semiconductors, contact resistance, contact functionalization, bending stability, bias-stress stability, long-term air stability

## Abstract

Three promising small-molecule organic semiconductors, namely, 2,9-bis­(heptafluoropropyl)-4,7,11,14-tetrabromo-1,3,8,10-tetraazaperopyrene (TAPP-Br_4_), *N,N′*-bis­(2,2,3,3,4,4,4-fluorobutyl)-(1,7 and 1,6)-dicyano-perylene-tetracarboxylic diimide (ActivInk N1100), and diphenylethyl-3,4,9,10-benzo­[*de*]­isoquinolino­[1,8-*gh*]­quinolinetetracarboxylic diimide (PhC_2_-BQQDI), are employed for the fabrication of *n*-channel thin-film transistors (TFTs). The TFTs are fabricated on rigid silicon and flexible polymeric substrates, in both the inverted staggered (bottom-gate, top-contact) and the inverted coplanar (bottom-gate, bottom-contact) device architecture. In the coplanar TFTs, the source and drain contacts are functionalized with one of four different thiols to minimize contact resistance. The best performance is obtained for PhC_2_-BQQDI TFTs with bottom contacts functionalized using 4-(methylsulfanyl)-thiophenol (MeSTP). For these TFTs, we measure contact resistances of 130 Ω cm for TFTs fabricated on silicon substrates and 210 Ω cm for TFTs on flexible polymeric substrates; these are the smallest contact resistances reported to date for *n*-channel organic TFTs, despite the fact that the measurements are carried out in ambient air. The observation that functionalizing the contacts with MeSTP leads to the smallest contact resistance is consistent with the fact that MeSTP provides the lowest effective work function. The TFTs fabricated on flexible polymeric substrates showed excellent bending stability.

## Graphical abstract

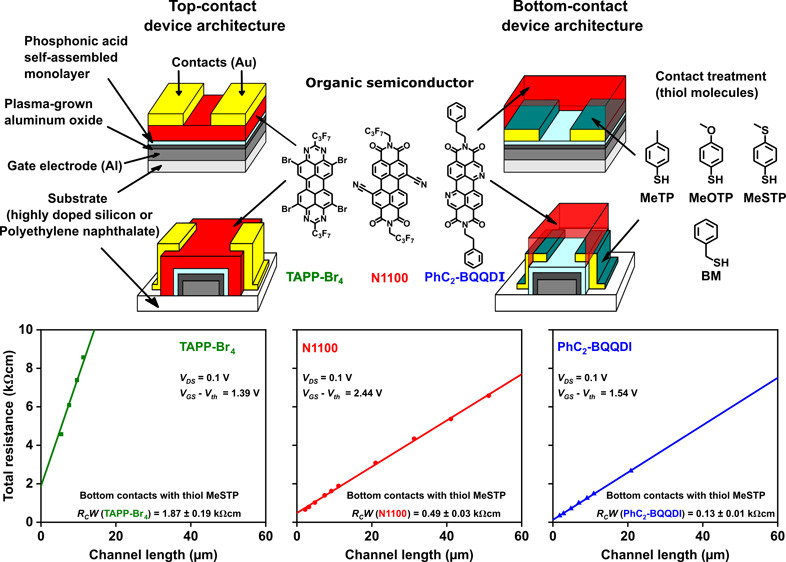

## Introduction

Thin-film transistors (TFTs) based on organic semiconductors can be manufactured at relatively low process temperatures, making it possible to fabricate them not only on rigid substrates, such as silicon or glass, but also on flexible polymeric substrates and fabrics. This enables wearable electronics,^1^ sensors,^2^ radio-frequency identification (RFID) tags, and flexible displays.^3,4^ For mobile devices that rely on batteries, a low operating voltage and a low power consumption are critical requirements.^5,6^ The most effective approach to minimize the power consumption of integrated circuits is to combine p-channel and n-channel transistors in a complementary circuit topology.^5,6^

For flexible p-channel organic TFTs, charge-carrier mobilities in excess of 10 cm^2^ V^–1^ s^–1^,^7,8^ subthreshold swings at the physical limit (59 mV decade^–1^),^9^ contact resistances as small as 10 Ω cm,^7,10^ and excellent long-term stability have already been reported.^11^ In contrast, the performance of air-stable n-channel organic TFTs is still comparatively poor: The largest charge-carrier mobilities reported for flexible air-stable n-channel organic TFTs are no greater than about 1 cm^2^ V^–1^ s^–1^,^12–15^ and the steepest subthreshold swing reported to date is 80 mV decade^–1^.^16^

Perhaps more troubling, the lowest contact resistances reported so far for n-channel organic TFTs are 0.3 kΩ cm for TFTs on rigid substrates operated in a glovebox,^17^ 1.2 kΩ cm for TFTs on rigid substrates operated in air,^200^ 1.0 kΩ cm for flexible TFTs operated in a glovebox,^19^ and 15 kΩ cm for flexible TFTs operated in air.^20^ Such large contact resistances are problematic, as they restrict the dynamic TFT performance and thus the dynamic performance of organic complementary circuits.^21^ Indeed, the smallest signal propagation delays reported to date for flexible organic complementary ring oscillators are more than an order of magnitude larger than the smallest signal delays reported for unipolar ring oscillators based on p-channel organic TFTs.^10,22,23^

The fact that the performance and stability of n-channel organic TFTs are generally inferior to those of p-channel organic TFTs is related to the fact that the energy difference between the charge-transport level and the vacuum level is substantially smaller in n-channel TFTs than in p-channel TFTs. In p-channel organic TFTs, charge transport occurs in the highest occupied molecular orbital (HOMO), which typically has an energy of approximately −5.5 eV,^24^ whereas in n-channel TFTs, transport occurs in the lowest unoccupied molecular orbital (LUMO), which usually has an energy of about −4.0 eV.^25^ The smaller energy difference between the transport level and the vacuum level makes the charge carriers in n-channel organic TFTs more susceptible to oxygen- and water-induced destabilization and trapping,^26^ which results in inferior charge-carrier mobility and stability compared to p-channel organic TFTs. Therefore, the performance and stability of n-channel organic TFTs generally benefit from a low-lying LUMO level of the semiconductor, ideally below −4.0 eV.^26^

Successful approaches to the synthesis of organic semiconductors with a low-lying LUMO level include the incorporation of nitrogen atoms into the molecular backbone (i.e., the design of N-heteropolycycles) and the addition of strongly electron-withdrawing substituents.^25^ This report focuses on three promising vacuum-deposited small-molecule N-heteropolycycles with low-lying LUMO levels, namely, 2,9-bis­(heptafluoropropyl)-4,7,11,14-tetrabromo-1,3,8,10-tetraazaperopyrene (TAPP-Br_4_; first reported by Geib et al. in 2013),^27^ *N*,*N′*-bis­(2,2,3,3,4,4,4-fluorobutyl)-(1,7 and 1,6)-dicyano-perylene-tetracarboxylic diimide (ActivInk N1100; first reported by Jones et al. in 2004),^28^ and diphenylethyl-3,4,9,10-benzo­[*de*]­isoquinolino­[1,8-*gh*]­quinolinetetracarboxylic diimide (PhC_2_-BQQDI; first reported by Okamoto et al. in 2020).^15^ These semiconductors have LUMO energies between −4.0 and −4.6 eV and have previously shown effective charge-carrier mobilities between 0.03 and 1.3 cm^2^ V^–1^ s^–1^ in flexible n-channel TFTs operated in air.^15,20,27^

Using these three semiconductors, we have fabricated low-voltage n-channel organic TFTs both on rigid silicon and on flexible polymeric substrates and both in the inverted staggered (bottom-gate, top-contact) and in the inverted coplanar (bottom-gate, bottom-contact) device architecture. For all TFTs, gold was used for the source and drain contacts. When fabricating organic TFTs with bottom contacts, the surface of the source and drain contacts can be functionalized with a chemisorbed thiol monolayer to minimize the contact resistance.^29^ For p-channel organic TFTs, pentafluorobenzenethiol (PFBT) is often used for this purpose,^10,30^ while for n-channel organic TFTs, methylthiophenol (MeTP), methoxythiophenol (MeOTP), and methylsulfanylthiophenol (MeSTP) have previously shown promising results.^31^ For the bottom-contact TFTs investigated here, we have therefore employed MeTP, MeOTP, and MeSTP, as well as benzyl mercaptan (BM) for comparison.^16^

The best device performance was obtained for bottom-contact TFTs using PhC_2_-BQQDI as the semiconductor and MeSTP for contact functionalization. For TFTs with this combination of device architecture and functional materials, we measured a contact resistance of 130 Ω cm for TFTs fabricated on silicon substrates and 210 Ω cm for TFTs on flexible polymeric substrates. These are the smallest contact resistances reported to date for n-channel organic TFTs, despite the fact that the measurements were carried out in ambient air. The flexible bottom-contact PhC_2_-BQQDI TFTs have an intrinsic channel mobility of 0.95 cm^2^ V^–1^ s^–1^, effective charge-carrier mobilities as high as 0.6 cm^2^ V^–1^ s^–1^, and a subthreshold swing as small as 77 mV decade^–1^. This is the smallest subthreshold swing reported to date for flexible n-channel organic TFTs operated in ambient air.

## Results and Discussion

### TFTs on Silicon Substrates

TFTs based on all three semiconductors (TAPP-Br_4_, N1100, and PhC_2_-BQQDI) were fabricated on silicon substrates, both in the top-contact and in the bottom-contact device architecture (see Figure 1).

**Figure 1:**
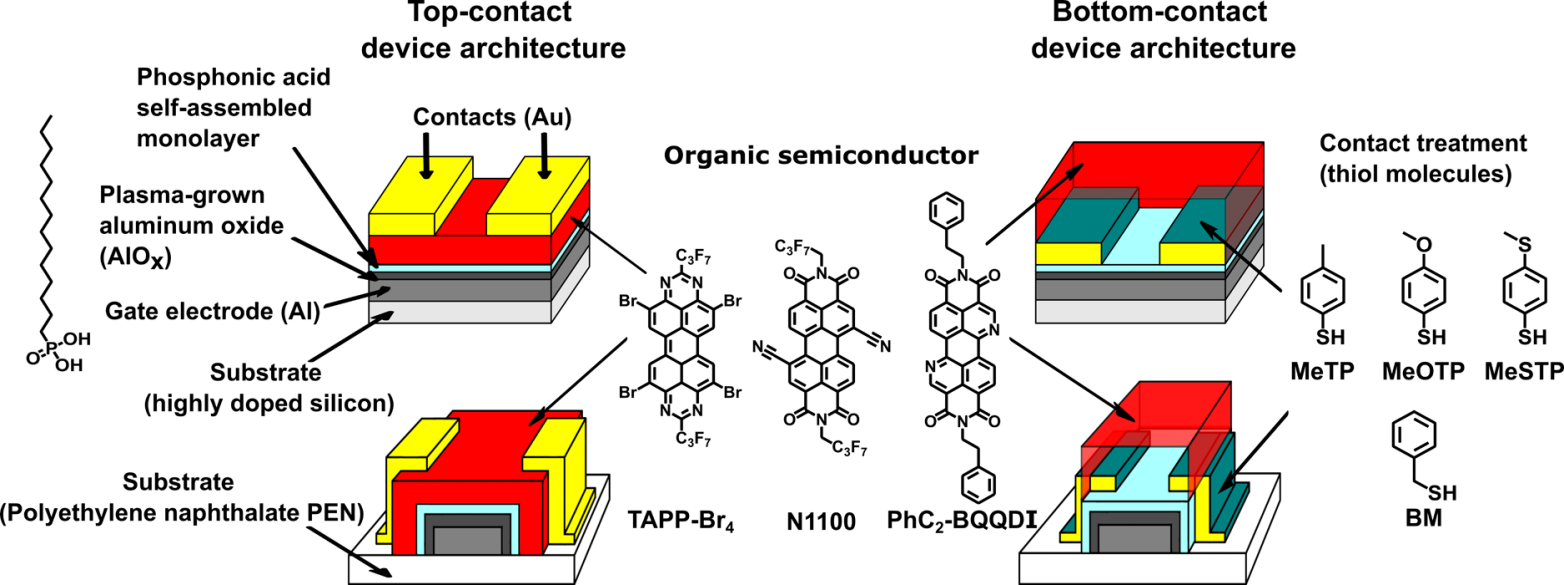
Schematic cross sections of the TFTs (left: top-contact device architecture; right: bottom-contact device architecture) and chemical structures of the organic semiconductors (TAPP-Br_4_, N1100, PhC_2_-BQQDI) and of the molecules used for the contact functionalization (MeTP, MeOTP, MeSTP, BM).

Measured transfer characteristics of TFTs fabricated on silicon substrates are shown in Figure 2 (top-contact TFTs) and Figure 3 (bottom-contact TFTs), output characteristics are shown in Figure S1, and the performance parameters extracted from the current–voltage characteristics are summarized in Table S1. The gate dielectric of the TFTs has a unit-area capacitance of 0.6 μF cm^–2^ (see Figure S2). All measurements were performed in ambient air.

**Figure 2:**
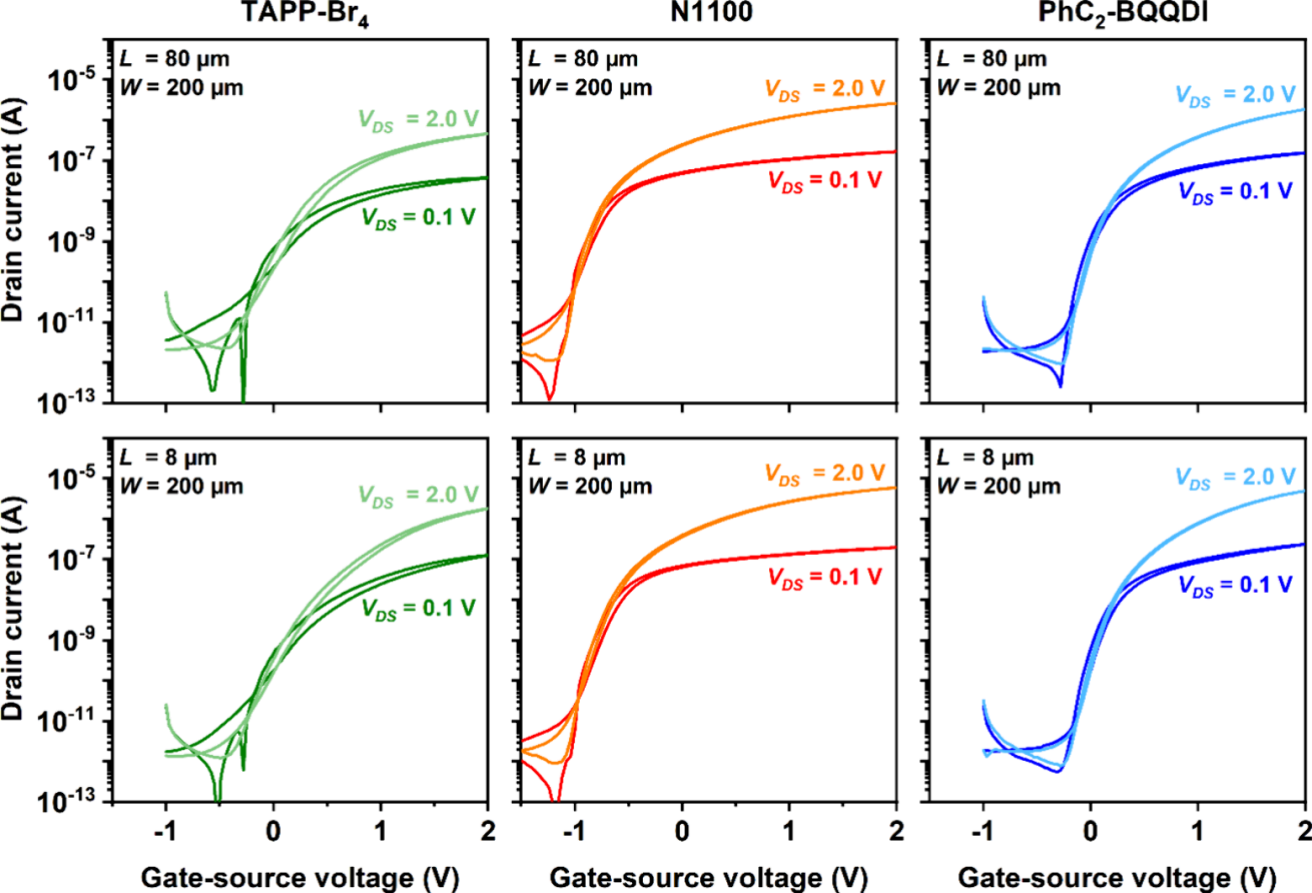
Transfer characteristics of top-contact TFTs fabricated on silicon substrates using all three semiconductors (TAPP-Br_4_, N1100, and PhC_2_-BQQDI). The TFTs have a channel length (*L)* of 80 μm (top row) or 8 μm (bottom row).

**Figure 3:**
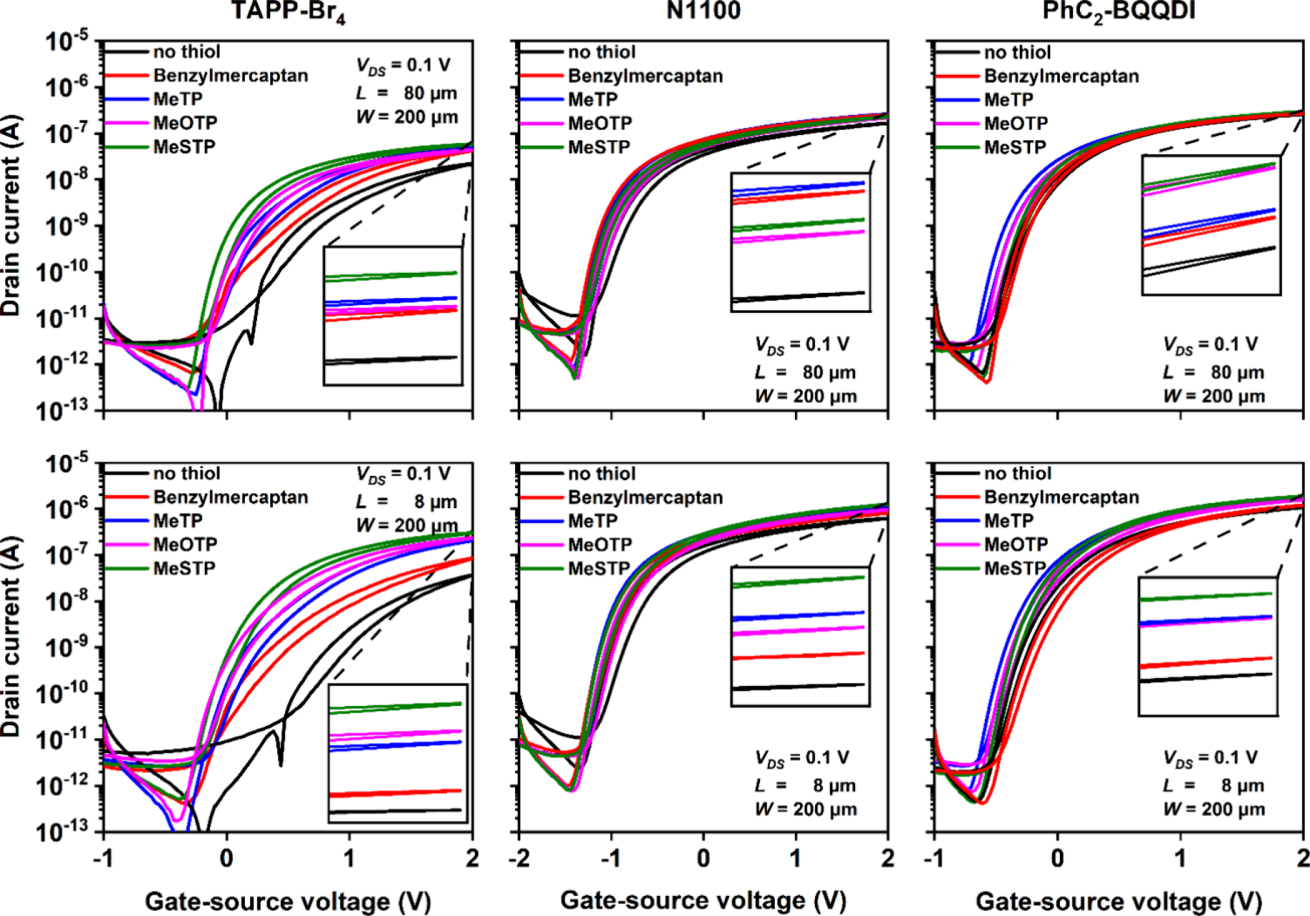
Transfer characteristics of bottom-contact TFTs fabricated on silicon substrates using all three semiconductors (TAPP-Br_4_, N1100, and PhC_2_-BQQDI) and either MeTP, MeOTP, MeSTP, or BM for the contact functionalization. The TFTs have a channel length (*L*) of 80 μm (top row) or 8 μm (bottom row).

The effective charge-carrier mobility (*μ_eff_*) and the threshold voltage (*V_th_*) of the TFTs were extracted from the measured transfer characteristics by fitting the measurement data to the current–voltage equations for the saturation regime and the linear regime of operation:^32–34^

$$I_{D}=\frac{\mu_{\mathrm{eff},\mathrm{sat}}\cdot C_{\mathrm{diel}}\cdot W}{2L}(V_{\mathrm{GS}}-V_{\mathrm{th}}{)}^{2}\;\mathrm{for}\;V_{\mathrm{DS}}>V_{\mathrm{GS}}-V_{\mathrm{th}}>0\;(\mathrm{saturation}\;\mathrm{regime})$$ 1

$$I_{D}=\frac{\mu_{\mathrm{eff},\mathrm{lin}}\cdot C_{\mathrm{diel}}\cdot W}{L}\left[(V_{\mathrm{GS}}-V_{\mathrm{th}})\cdot V_{\mathrm{DS}}-{\frac{V_{\mathrm{DS}}}{2}}^{2}\right]\;\mathrm{for}\;V_{\mathrm{GS}}-V_{\mathrm{th}}>V_{\mathrm{DS}}\;(\mathrm{linear}\;\mathrm{regime})$$ 2

where *μ_eff,sat_* is the effective charge-carrier mobility in the saturation regime, *μ_eff,lin_* is the effective charge-carrier mobility in the linear regime, *I*_*D*_ is the drain current, *W* is the channel width, *L* is the channel length, *C_diel_* is the gate-dielectric capacitance per unit area (0.6 μF cm^–2^), *V*_GS_ is the gate-source voltage, *V_th_* is the threshold voltage, and *V_DS_* is the drain-source voltage.

One observation in Figures 2 and 3 is that the turn-on voltage of the N1100 TFTs (−1.1 to −1.5 V, depending on the device architecture and the choice of thiol for the contact functionalization) is systematically more negative than that of the TAPP-Br_4_ and PhC_2_-BQQDI TFTs (−0.2 to −0.7 V). A possible explanation is that N1100 has a larger electron affinity than TAPP-Br_4_ and PhC_2_-BQQDI, which has been reported to lead to a more negative turn-on voltage.^35,36^ In principle, the turn-on voltage can be shifted toward more positive values by employing a fluoroalkyl self-assembled monolayer (SAM) instead of an alkyl SAM as part of the gate dielectric.^5^ However, for TAPP-Br_4_ and PhC_2_-BQQDI, the alkyl SAM is apparently the better choice, given that for these two semiconductors, the alkyl SAM produces a turn-on voltage close to zero.

The top-contact TFTs (Figure 2) have effective charge-carrier mobilities (μ_eff,sat_) close to 0.2 cm^2^ V^–1^ s^–1^ for TAPP-Br_4_, 0.3 cm^2^ V^–1^ s^–1^ for N1100, and 0.7 cm^2^ V^–1^ s^–1^ for PhC_2_-BQQDI, all for a channel length of 80 μm (see Table S1). For the bottom-contact TFTs (Figure 3), it is evident that the effective charge-carrier mobility depends on the choice of the thiol that is used to functionalize the surface of the source and drain contacts prior to the organic-semiconductor deposition. The reason is that the effective charge-carrier mobility depends on the contact resistance, which in turn depends on the choice of thiol, as discussed below. Atomic force microscopy (AFM) images of Au source and drain contacts with and without thiol functionalization are shown in Figure S3; the analysis of these AFM images indicates a root-mean-square surface roughness of 2 nm, regardless of whether or not the gold is functionalized with a thiol.

For all three semiconductors and all four thiols (MeTP, MeOTP, MeSTP, and BM) employed here, the effective charge-carrier mobility improves (if only slightly) upon contact functionalization compared to bottom-contact devices without functionalization, regardless of the choice of thiol. With the best-performing thiol, the effective charge-carrier mobilities (*μ_eff,lin_*) of the bottom-contact TFTs with a channel length of 80 μm are 0.1 cm^2^ V^–1^ s^–1^ for TAPP-Br_4_, 0.5 cm^2^ V^–1^ s^–1^ for N1100, and 0.9 cm^2^ V^–1^ s^–1^ for PhC_2_-BQQDI (see Table S1). In other words, for N1100 and PhC_2_-BQQDI, the effective charge-carrier mobility is slightly larger in the bottom-contact TFTs than in the top-contact TFTs, whereas for TAPP-Br_4_, the effective charge-carrier mobility is slightly larger for the top-contact architecture than for the bottom-contact architecture.

To determine the contact resistance of the TFTs, we applied the transfer length method (TLM).^9,30^ Despite the fact that the accuracy of the TLM is compromised by a number of assumptions and simplifications,^37^ it is by far the most widely utilized method to determine the contact resistance of organic TFTs and thus facilitates the most meaningful comparison with literature results.^38,39^ For this purpose, the transfer characteristics of TFTs with channel lengths ranging from 1 to 100 μm were measured at a drain-source voltage of 0.1 V (linear regime of operation).

To improve the reliability of the TLM analysis, we determined the actual channel lengths of all TFTs from scanning electron microscopy (SEM) images (Figure S4) rather than relying on the nominal channel lengths for the TLM analysis. For the TFTs discussed here, which were fabricated by stencil lithography, we found that the actual channel length is larger by approximately 0.7 μm than the nominal channel length. From the measured transfer characteristics of the TFTs, the total TFT resistance is calculated and is then treated as the sum of the contact resistance and the channel resistance. When the channel-width-normalized total resistance *R·W* is plotted as a function of the channel length, the width-normalized contact resistance *R*_*C*_ *·W* can be extracted by extrapolating the data according to the relation between the total resistance and the channel length to a channel length of zero.

The intrinsic channel mobility μ_0_ can be obtained either from the slope of the TLM fit (i.e., from the sheet resistance of the semiconductor; eq 5) or from the channel-length dependence of the effective charge-carrier mobility (eq 6):

$$R=R_{C}+R_{\mathrm{channel}}=\frac{V_{DS}}{I_{D}}$$ 3

$$R\cdot W=R_{C}\cdot W+R_{\mathrm{sheet}}\cdot L$$ 4

$$R_{\mathrm{sheet}}=\frac{R_{\mathrm{channel}}\cdot W}{L}=\frac{1}{\mu_{0}\cdot C_{\mathrm{diel}}\cdot (V_{\mathrm{GS}}-V_{\mathrm{th}})}$$ 5

$$\mu_{\mathrm{eff},\mathrm{lin}}=\frac{\mu_{0}}{1+\frac{L_{1/2}}{L}}$$ 6

where *R* is the total resistance, *R*_*C*_ is the contact resistance, *R*_channel_ is the channel resistance, *R*_sheet_ is the sheet resistance of the semiconductor layer, μ_eff,lin_ is the effective charge-carrier mobility in the linear regime of operation, μ_0_ is the intrinsic channel mobility, and *L*_1/2_ is a characteristic channel length at which the contact resistance equals the channel resistance.^40^

In eq 5, the expression *C*_diel_·(*V*_*GS*_ *-V*_*th*_) describes the mobile charge density in the channel. The accuracy of this expression depends on a number of assumptions. According to Horowitz et al.,^41^ the total charge density is *Q*_*tot*_ = *C* *_diel_·(V_GS_‑V_FB_)*, where *Q*_tot_ is the sum of the mobile charge density and the trapped charge density, and *V_FB_* is the flat-band voltage. If we ignore the trapped charges and assume that the threshold voltage is equal to the flat-band voltage, we get *Q*_*mob*_ = *C*_*diel*_·(*V_GS_*-*V_th_*), so in this case, the accuracy of the expression depends on how large the trapped charge density is and how much the threshold voltage deviates from the flat-band voltage. According to Marinov et al.,^42^ the charge density is *Q*_*x*_ = *C* *_diel_·*(*V_GS_*-*V_th_*–*V*_*x*_), where *Q*_*x*_ and *V*_*x*_ are the charge density and the potential at point *x* along the channel. If we assume that the drain-source voltage is close to zero, we get *Q*_*mob*_ = *C*_*diel*_·(*V*_*GS*_-*V*_th_), so in this case, the accuracy of the expression depends on how large the applied drain-source voltage is.

The results of the TLM analysis of the TFTs fabricated on silicon substrates are summarized in Figure 4 and Figures S5, S6, S7, and S8 and Table 1.

**Figure 4:**
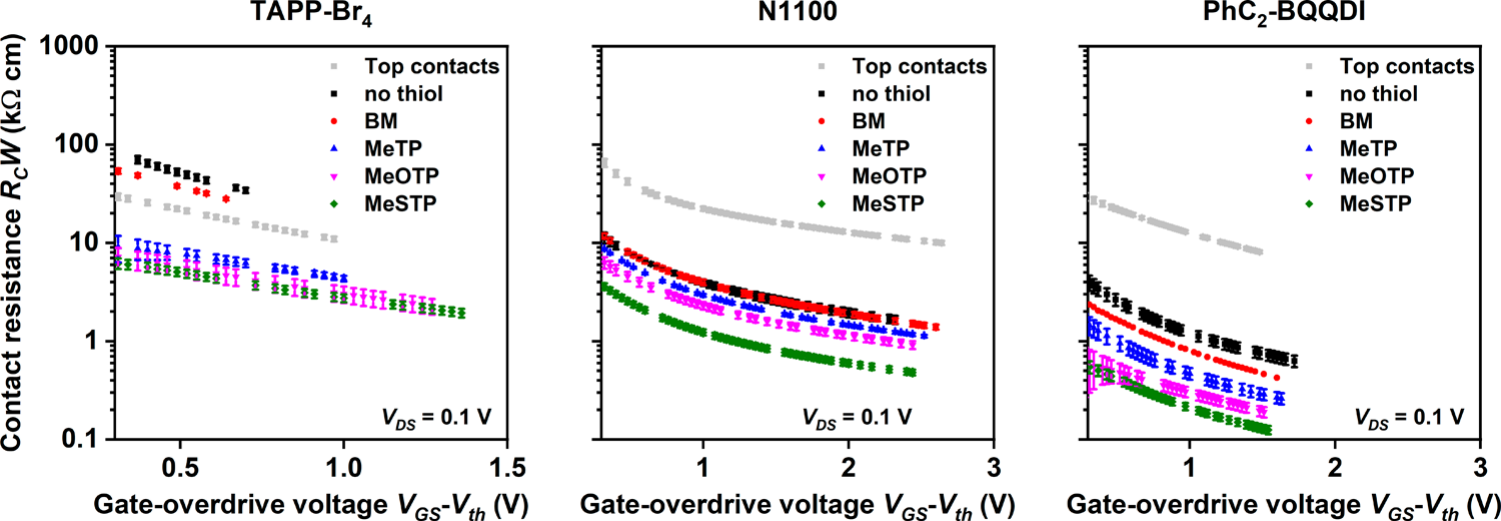
Contact resistance (*R*_*C*_ *W*) of top-contact and bottom-contact TFTs fabricated on silicon substrates using all three semiconductors (TAPP-Br_4_, N1100, and PhC_2_-BQQDI) and either MeTP, MeOTP, MeSTP, or BM for the contact functionalization in the bottom-contact TFTs, extracted using the transfer length method and plotted as a function of the gate-overdrive voltage (*V*_GS_-*V*_th_). The minimum contact resistances (at the highest gate-overdrive voltages) extracted from these measurements are summarized in Table 1. For all three semiconductors, the smallest contact resistance is obtained with the MeSTP-functionalized bottom contacts.

**Table 1:** Intrinsic Channel Mobility (*μ*_*0*_) and Contact Resistance (*R_C_W*) of Top-Contact and Bottom-Contact TFTs Fabricated on Silicon Substrates Using All Three Semiconductors and All Four Thiols, Extracted Using the Transfer Length Method at the Largest Gate-Overdrive Voltage (*V*_GS_-*V*_th_) in the Linear Regime of Operation (*V*_DS_ = 0.1 V, *V*_GS_ = 2 V) and Averaged Over the Number of TFTs Given in Parentheses

semiconductor	device architecture/thiol	maximum intrinsic channel mobility μ*_0_*	average intrinsic channel mobility μ*_0_*	minimum contact resistance *R* *_C_* *W*	average contact resistance *R* *_C_* *W*
		(cm^2^ V^–1^ s^–1^)	(cm^2^ V^–1^ s^–1^)	(kΩ cm)	(kΩ cm)
TAPP-Br_4_	TC/none (6)	0.32	0.20 ± 0.06	11	19 ± 6
	BC/None (2)	0.21	0.18 ± 0.04	35	41 ± 9
	BC/BM (3)	0.39	0.27 ± 0.12	4.8	21 ± 14
	BC/MeTP (3)	0.25	0.20 ± 0.06	3.6	6.9 ± 4.9
	BC/MeOTP (4)	0.23	0.15 ± 0.07	2.3	6.7 ± 4.9
	BC/MeSTP (4)	0.23	0.16 ± 0.07	1.9	4.3 ± 3.4
N1100	TC/none (3)	1.0	0.78 ± 0.26	10	14 ± 5
	BC/none (3)	0.80	0.65 ± 0.25	1.7	2.1 ± 0.4
	BC/BM (3)	0.65	0.52 ± 0.16	1.4	2.4 ± 1.7
	BC/MeTP (3)	0.75	0.57 ± 0.18	1.1	1.4 ± 0.3
	BC/MeOTP (8)	0.64	0.50 ± 0.12	0.93	1.3 ± 0.3
	BC/MeSTP (8)	0.66	0.52 ± 0.09	0.49	1.1 ± 0.5
PhC_2_-BQQDI	TC/none (4)	1.4	1.2 ± 0.5	8.1	8.6 ± 0.6
	BC/none (6)	1.0	0.89 ± 0.11	0.63	2.7 ± 1.9
	BC/BM (8)	1.1	0.93 ± 0.09	0.42	1.5 ± 0.9
	BC/MeTP (6)	1.1	0.95 ± 0.10	0.26	0.9 ± 0.6
	BC/MeOTP (8)	1.0	0.86 ± 0.11	0.19	1.1 ± 0.7
	BC/MeSTP (16)	1.1	0.82 ± 0.16	0.13	0.8 ± 0.4

The contact resistances of the top-contact TFTs are quite similar for all three semiconductors, with minimum values of 11 kΩ cm for TAPP-Br_4_, 10 kΩ cm for N1100, and 8 kΩ cm for PhC_2_-BQQDI. These values are close to the lowest contact resistances reported in literature for top-contact PhC_2_-BQQDI and N1100 TFTs.^20,43^ There is no previous report on the contact resistance of TAPP-Br_4_ transistors.

The contact resistances of the bottom-contact TFTs with appropriate contact functionalization are significantly smaller, with minimum values of 1.9 kΩ cm for TAPP-Br_4_, 0.49 kΩ cm for N1100, and 0.13 kΩ cm for PhC_2_-BQQDI. The contact resistance of 0.13 kΩ cm obtained for the bottom-contact PhC_2_-BQQDI TFTs with the MeSTP-functionalized contacts is the smallest contact resistance reported to date for n-channel organic TFTs. The fact that the bottom-contact TFTs have a smaller contact resistance than the top-contact TFTs is consistent with drift-diffusion simulations showing that bottom-contact TFTs can outperform otherwise equivalent top-contact TFTs in terms of contact resistance, provided the gate dielectric is sufficiently thin.^44,45^ For p-channel organic TFTs, these predictions have been verified experimentally;^29,46^ for n-channel organic TFTs, the results shown here provide to our knowledge the first experimental confirmation.

While our bottom-contact TFTs outperform the top-contact TFTs in terms of contact resistance, they have a smaller intrinsic channel mobility (*μ_0_)* than the top-contact TFTs (e.g., for PhC_2_-BQQDI, the intrinsic channel mobility reaches 1.4 cm^2^ V^–1^ s^–1^ in top-contact TFTs, but only 1.1 cm^2^ V^–1^ s^–1^ in bottom-contact TFTs; see Table 1). A similar behavior was previously observed for p-channel organic TFTs.^46^ A possible explanation is that in top-contact TFTs, the semiconductor is deposited onto a smooth and homogeneous gate-dielectric surface, whereas in bottom-contact TFTs, the thin-film morphology of the organic-semiconductor layer is disturbed by the topography and the surface-energy contrast imposed by the presence of the source and drain contacts. In other words, while the bottom-contact device architecture is helpful in reducing the contact resistance, it affects the semiconductor morphology and thereby the intrinsic channel mobility (*μ*_0_). Nevertheless, for improved dynamic TFT performance, a smaller contact resistance is usually more beneficial than a larger intrinsic channel mobility.^46^

Regardless of the semiconductor, the contact resistance of the bottom-contact TFTs depends strongly on the contact functionalization (e.g., for PhC_2_-BQQDI: 0.13 kΩ cm with MeSTP, 0.43 kΩ cm with BM), while the intrinsic channel mobility (*μ*_0_) is not affected significantly by the thiol functionalization of the contacts (see Table 1). Without thiol, the contact resistance is rather large (for TAPP-Br_4_, it is even larger than in the top-contact TFTs). The thiol that provides the smallest contact resistance for all three semiconductors is MeSTP, followed by MeOTP, MeTP, and BM.

According to the thermionic-emission model, the efficiency of the charge transfer between the source/drain contacts and the semiconductor depends exponentially on the height of the Schottky barrier at the semiconductor-metal interface.^30,32^ The general design principles given by the Schottky-Mott rule yield the Schottky-barrier height as the energy difference between the charge-transport level of the semiconductor (the LUMO in n-channel TFTs) and the Fermi level of the source and drain contacts, and this difference should ideally be as small as possible to minimize the contact resistance.^32^ However, when an organic semiconductor is brought into contact with a metal, the electronic interactions between the metal and the semiconductor induce a density of gap states in the semiconductor near the interface with the metal. These metal-induced gap states prevent the Fermi level from moving through the HOMO–LUMO gap of the semiconductor, and as a result of this Fermi-level pinning, the Schottky-barrier height becomes independent of the energy difference between the charge-transport level of the semiconductor and the Fermi level of the contacts.^32,47^ In other words, the influence of the metal work function on the Schottky-barrier height can be smaller than predicted by the Schottky-Mott rule, or even nonexistent. To derive a general design principle for the fabrication of organic TFTs, it can nevertheless be useful to estimate the Schottky-barrier height for the various combinations of semiconductor and functionalized source and drain contacts. The LUMO energy of the semiconductor can be estimated from electrochemical measurements and density functional theory (DFT) calculations, while the work function of the (functionalized) contacts can be determined by ultraviolet photoelectron spectroscopy (UPS).

The electrochemical properties of the three organic semiconductors were evaluated by performing *in situ* spectroelectrochemical measurements. This approach not only makes it possible to identify the redox species during electrochemical reduction oroxidation, but it can also be applied to determine the frontier-orbital energy levels by evaluating the spectral evolution of the neutral and the first reduced or the first oxidized states (to determine the energy of the LUMO and the HOMO, respectively).^48–50^

For TAPP-Br_4_ and PhC_2_-BQQDI, electrochemical measurements were performed on vacuum-deposited thin-films in an acetonitrile/TBAPF_6_ electrolyte. For N1100, thin-film measurements were unsuccessful, since the N1100 films immediately dissolved from the working electrode upon immersion into the acetonitrile electrolyte. The electrochemical measurements on N1100 were therefore performed in solution by dissolving N1100 in the electrolyte (dichloromethane/TBAPF_6_). To provide an environment resembling (at least remotely) that of a thin film, we performed the measurements in a custom-built electrochemical cell in which the distance between the working electrode (a Pt disc) and the bottom of the electrochemical cell can be made extremely small (below 20 μm), thereby effectively confining the semiconductor into a thin layer (thin-layer conditions). The cyclic-voltammetry (CV) curves of the three organic semiconductors are shown in Figure 5A–C. TAPP-Br_4_ and N1100 show a clear 2-fold reduction process in the forward scan, which can be attributed to the reduction of the neutral state to a first reduced and a second reduced state. In the case of N1100, the reduction appeared to be fully reversible. In accordance with literature, this involves radical anion and dianion redox species.^15,27,51,52^ The CV of PhC_2_-BQQDI shows only one large redox wave in the forward scan and two small redox waves in the backward scan, suggesting a two-step reduction process for this semiconductor, as well.

**Figure 5:**
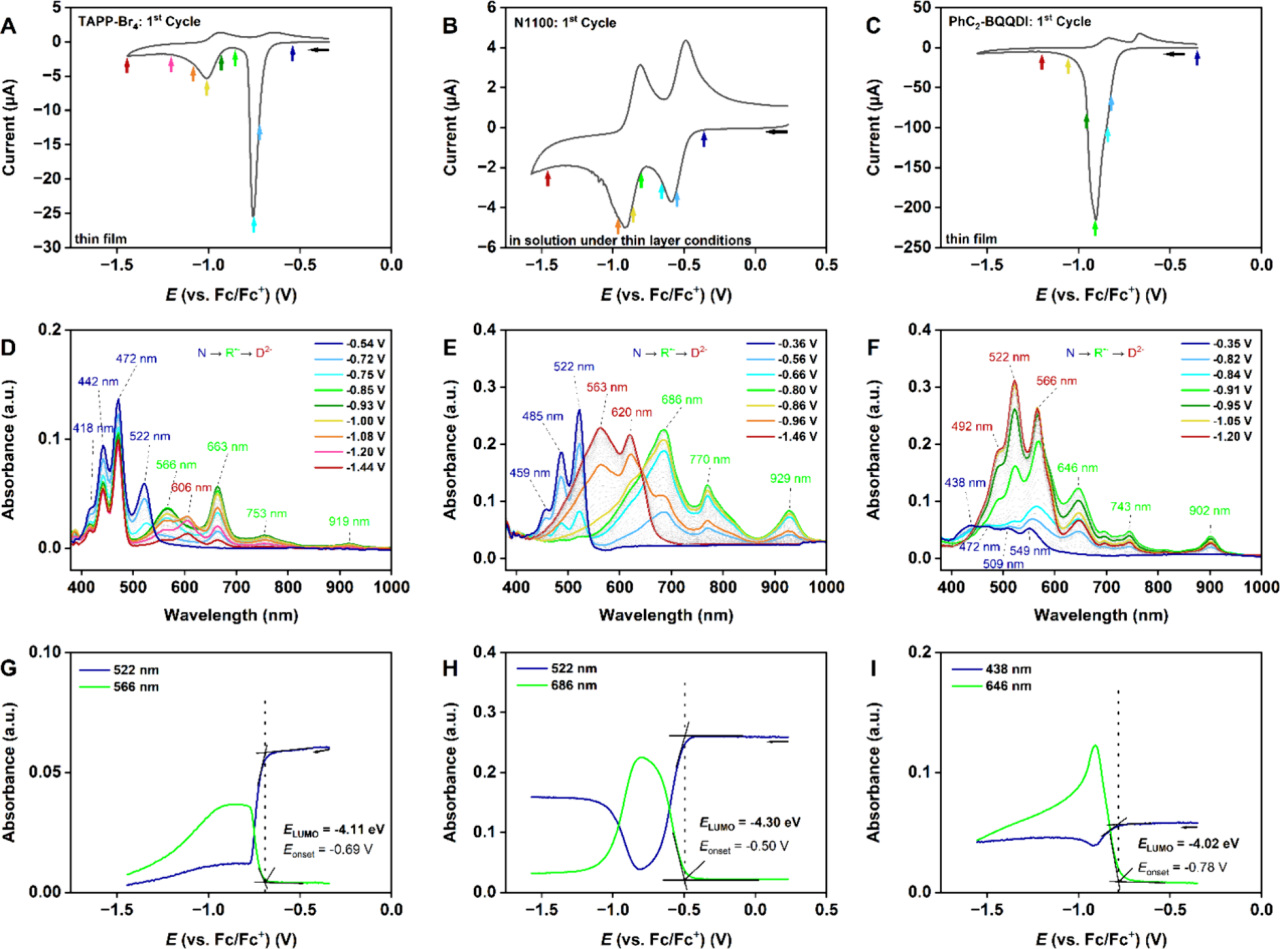
Results of *in situ* spectroelectrochemical measurements performed on vacuum-deposited thin-films of TAPP-Br_4_ (left) and PhC_2_-BQQDI (right) in a 0.1 M TBAPF_6_/MeCN electrolyte and on N1100 (center) dissolved in a 0.1 M TBAPF_6_/DCM electrolyte under thin-layer conditions. The measurements were performed at a scan rate of 20 mV s^–1^. The colors of the data points in panels (A–C) indicate the potential at which the UV–vis spectra that are shown in panels (D–F) were recorded. UV–vis spectra were recorded during the forward scan of the cyclic-voltammetry measurement. (G–I) Absorbance evolution at characteristic wavelengths for the neutral state (dark blue N) and the first reduced state (light green R^•–^) as a function of the applied potentials. From the spectral onsets, a reduction onset is obtained, which is then used to calculate the LUMO energy. The results are summarized in Tables 2 and 3 .

Figure 5D–F shows the corresponding *in situ* UV–vis spectra, which were recorded at the indicated potentials in the forward scan of the CV cycles. The characteristic bands obtained for the different reduced states are summarized in Table 2.

**Table 2:** Main Characteristic Bands for the Neutral, First Reduced, and Second Reduced State

semiconductor	neutral N (nm)	first reduced state R^•–^ (nm)	second reduced state D^2–^ (nm)
TAPP-Br_4_	418, 442, 472, 522	566, 663, 753, 919	606
N1100	459, 485, 522	686, 770, 929	563, 620
PhC_2_-BQQDI	438, 472, 509, 549	646, 743, 902	492, 522, 566

The UV–vis spectrum of the neutral TAPP-Br_4_ displays four vibronic bands of the π* ← π absorption at wavelengths of 418, 442, 472, and 522 nm (Figure 5D, dark blue N). In the potential range from −0.69 to −0.93 V, these bands continuously decreased, while four new bands at wavelengths of 566, 663, 753, and 919 nm developed that have their maximum absorbance at a potential of −0.85 V, with insignificant deviation for potentials up to −0.93 V. This redox state can be assigned to the first reduced radical anion state (Figure 5D, light green R^•–^). An isosbestic point can be detected at a wavelength of 540 nm. In the potential range from −0.95 to −1.44 V, the characteristic bands of the first reduced state decrease, and a new band appears at a wavelength of 606 nm that can be attributed to the second reduced state (dianion species), in accordance with the second redox wave in the cyclic voltammogram (Figure 5D, red D^2–^).

The UV–vis spectrum of the neutral state of N1100 displays three vibronic absorption bands at wavelengths of 459, 485, and 522 nm (Figure 5E, dark blue N). During the first reduction process in the potential range from −0.50 to −0.80 V, these bands decrease and almost disappear at a potential of −0.80 V, while three new bands at wavelengths of 686, 770, and 929 nm reach their maxima in absorbance (Figure 5E, light green R^•–^). We assign these new bands to the first reduced state of N1100. Further reduction leads to the development of two new bands at wavelengths of 563 and 620 nm in the potential range from −0.80 to −1.57 V, which are a clear indication of a second reduced state (Figure 5E, red D^2–^). Two isosbestic points are visible at wavelengths of 542 and 642 nm.

During the reduction of PhC_2_-BQQDI, the vibronic absorption bands of the neutral film at wavelengths of 438, 472, 509, and 549 nm not only increase in absorbance, but are also shifted to longer wavelengths, starting from a potential of −0.78 V (Figure 5F, dark blue N). In the potential range up to −0.91 V, new bands with maxima at wavelengths of 646, 743, and 902 nm appear. These can be assigned to the first reduced state (Figure 5F, light green R^•–^). Starting from a potential of −0.91 V, these bands decrease, while absorption bands at wavelengths of 492, 522, and 566 nm start to increase, suggesting the appearance of the second reduced state (Figure 5F, red D^2–^).

These data clearly demonstrate that spectral characterization coupled with CV allows us to distinguish the different redox states, which is not possible with CV only.

To determine the LUMO energies of the three organic semiconductors, we analyzed the evolution of the absorbance of the neutral and first reduced states during reduction (Figure 5G–I). In the case of TAPP-Br_4_, we used the characteristic bands at wavelengths of 522 nm for the neutral state and 566 nm for the first reduced state to obtain the reduction onset potential *E*_onset_ through the tangent method (Figure 5G). With *E*_onset_ = −0.69 V, a LUMO energy of −4.11 eV was calculated for TAPP-Br_4_ by assuming that the redox potential of the Fc/Fc^+^ redox couple is located at an energy of −4.8 eV on the Fermi scale.^53^

Using the same method, LUMO energies of −4.30 and −4.02 eV were obtained for N1100 and PhC_2_-BQQDI, respectively (Figure 5H,I). From the CV curves, we were able to determine the half-wave potentials of TAPP-Br_4_ and N1100, and by rescaling to the Fermi scale, LUMO energies of −4.10 eV (TAPP-Br_4_) and −4.26 eV (N1100) were obtained. Unfortunately, it was not possible to determine the half-wave potential of PhC_2_-BQQDI from the CV curve (Figure 5C).

The LUMO energies, including the half-wave potentials and spectral onsets of the three organic semiconductors, are summarized in Table 3. The values are similar to previously reported values.^15,27,52^ Since electron-withdrawing substituents such as cyano and alkyl halide groups can effectively lower the LUMO energy,^25^ the LUMO energies of TAPP-Br_4_ (−4.11 eV) and N1100 (−4.30 eV) are lower than that of PhC_2_-BQQDI (−4.02 eV). As mentioned in the Introduction, the performance and stability of n-channel organic TFTs generally benefit from a low-lying LUMO level of the semiconductor, ideally below −4.0 eV.^26,54^

**Table 3:** Lowest Unoccupied Molecular Orbital (LUMO) Energies Estimated Based on Cyclic-Voltammetry (CV) Measurements and Computed Using Density Functional Theory (DFT)

method	TAPP-Br_4_		N1100		BQQDI	
	*E*_1/2_ or *E*_onset_ (V)	*E*_LUMO_ (eV)	*E*_1/2_ or *E*_onset_ (V)	*E*_LUMO_ (eV)	*E*_*1/2*_ or *E*_onset_ (V)	*E*_LUMO_ (eV)
CV	–0.70	–4.10	–0.54	–4.26		Not possible (PhC_2_-BQQDI)
spectra	–0.69	–4.11	–0.50	–4.30	–0.78	–4.02 (PhC_2_-BQQDI)
DFT		–4.24		–4.62		–4.08 (Ethyl-BQQDI)

We also computed the LUMO energies of the three organic semiconductors using density functional theory. Details on the DFT calculations as well as the xyz-coordinates of the optimized structures (Table S3) are given in the Supporting Information. The results are summarized in Table 3.

The work functions of bottom source/drain contacts with and without functionalization with MeTP, MeOTP, MeSTP, and BM that have been reported in literature are listed in Table 4.^31,55^ Some of the observations mentioned above regarding how the contact resistance depends on the choice of thiol can indeed be explained by the values in Tables 3 and 4, although others remain ambiguous. For example, since the LUMO energy of PhC_2_-BQQDI (−4.02 to −4.08 eV) and TAPP-Br_4_ (−4.10 to −4.24 eV) is closest to the Fermi level of MeSTP-functionalized gold contacts (−4.19 eV), the contact resistance of TFTs based on these two semiconductors is indeed expected to be lower for MeSTP than for the other thiols (see Figure 6). For N1100, however, the LUMO energy (−4.26 to −4.62 eV) is closest to the Fermi level of MeTP or MeOTP (−4.28 and −4.24 eV, respectively) instead of MeSTP, and therefore, the Schottky barrier (and thus the contact resistance) would be expected to have the smallest value for those thiols, not for MeSTP.

**Table 4:** Effective Work Functions Determined by Ultraviolet Photoelectron Spectroscopy (UPS) Measurements on Bare Au Bottom Contacts and on Au Contacts Functionalized with MeTP, MeSTP, MeOTP, or BM Reported in Literature^31,55^

UPS	bare gold	MeTP	MeOTP	MeSTP	BM
Effective work function (eV)	4.75	4.28	4.24	4.19	4.20

**Figure 6:**
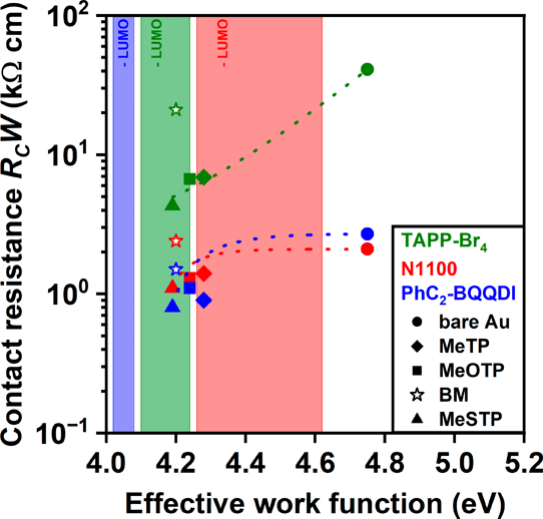
Contact resistance (average values) of bottom-contact TFTs fabricated on silicon substrates using all three semiconductors (TAPP-Br_4_, N1100, and PhC_2_-BQQDI) and either MeTP, MeOTP, MeSTP, or BM for the contact functionalization, plotted versus the effective work function of the thiol-functionalized Au contacts. The dependence of the effective work function on the choice of thiol is indicated in Table 3. The shaded regions represent the expected LUMO energy of the respective semiconductor (Table 2). The closed symbols represent MeSTP, MeOTP, MeTP, and bare Au (for increasing work function, i.e., from left to right), while the open star symbols represent benzyl mercaptan (BM). Since the contact resistance of the TFTs with the BM contact functionalization does not follow the trend (presumably due to the detrimental effect of the additional carbon atom between the anchor group (−SH) and the phenyl group), these data points are not included in the visualized tendencies indicated by the dashed lines. According to the Schottky-Mott rule, the smallest difference between the LUMO energy of the semiconductor and the effective work function of the contacts should lead to the smallest contact resistance, and for TAPP-Br_4_ and PhC_2_-BQQDI, this is indeed observed here.

Evidently, the energy difference between the charge-transport level of the semiconductor and the Fermi level of the source and drain contacts is not the only factor that determines the contact resistance of bottom-contact organic TFTs.^29,30,56^

The observation that among the thiols investigated here, benzyl mercaptan (BM) yields the largest contact resistance for all three semiconductors is possibly related to the fact that in the BM molecule, a methylene (−CH_2_−) unit is located between the anchor group (SH) and the phenyl ring. According to Heimel et al., alkylene spacer units located between the anchor group and the phenyl ring (as in BM) will suppress the electronic interactions (and thereby lead to an increase in the tunneling distance) between the metal and the phenyl ring.^57^ Although in the case of MeTP, MeOTP, and MeSTP, there are additional atoms located in the para position (i.e., above the phenyl ring; CH_3_ in the case of MeTP; O–CH_3_ in the case of MeOTP; and S-CH_3_ in the case of MeSTP), these atoms located above the phenyl ring have a smaller impact on the strength of the interaction between the metal and the organic-semiconductor layer.

### TFTs on Flexible PEN Substrates

In addition to TFTs on rigid silicon substrates, TFTs based on the semiconductors N1100 and PhC_2_-BQQDI were also fabricated on flexible polyethylene naphthalate (PEN) substrates, in both the top-contact and the bottom-contact device architecture. Figure S9 shows a photograph of TFTs fabricated on a flexible PEN substrate.

Since for the bottom-contact TFTs fabricated on silicon substrates, the best performance was obtained using MeSTP for the contact functionalization, this thiol was chosen for all bottom-contact TFTs fabricated on flexible PEN substrates. Transfer and output characteristics of the flexible TFTs are shown in Figure 7 and Figure S10, and the performance parameters extracted from the current–voltage characteristics are summarized in Table S2. As for the TFTs on silicon substrates, the gate dielectric has a unit-area capacitance of 0.6 μF cm^–2^, and all measurements were performed in ambient air.

**Figure 7:**
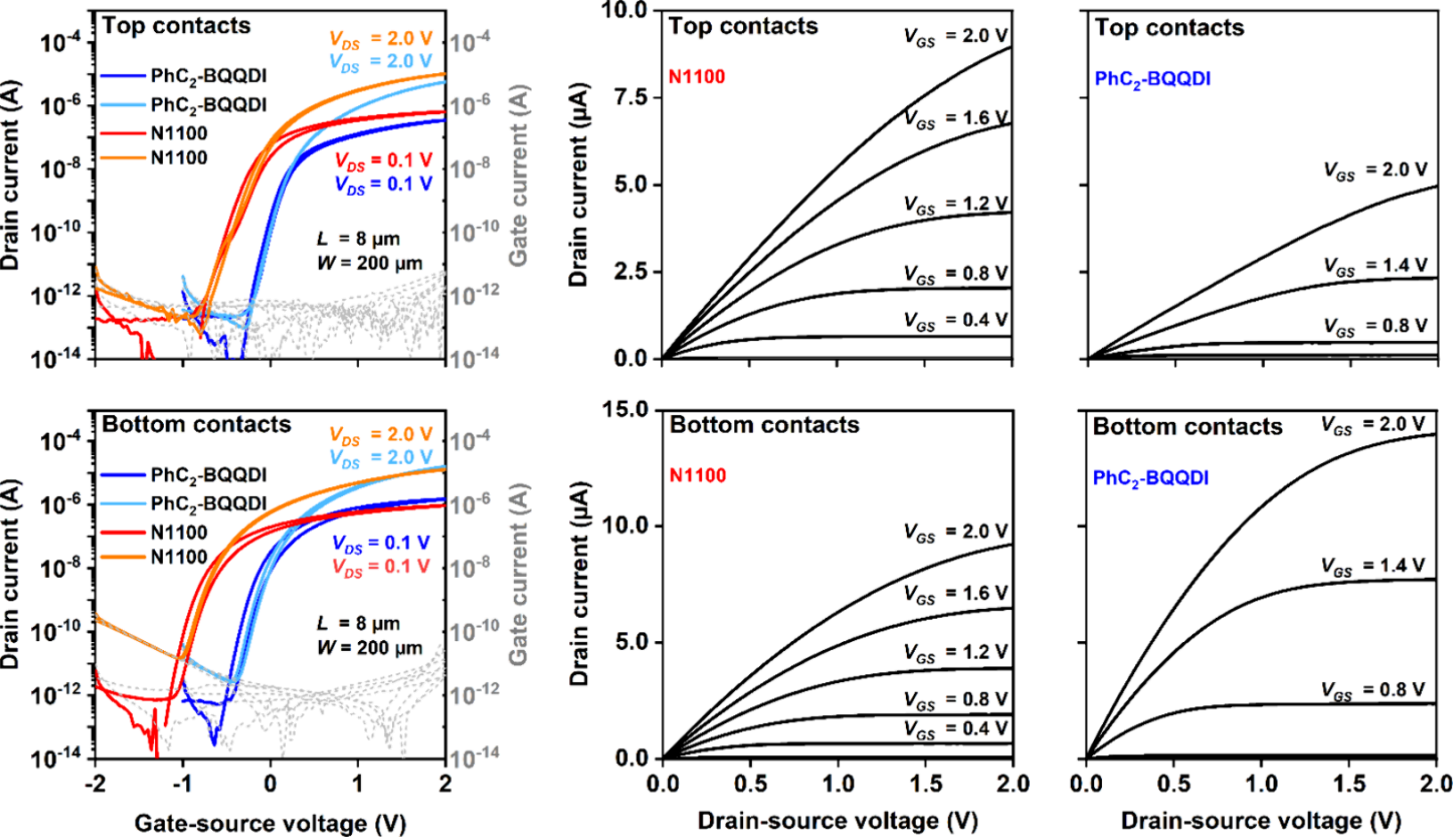
Transfer and output characteristics of top-contact (top row) and bottom-contact TFTs (bottom row) fabricated on flexible PEN substrates using the semiconductors N1100 and PhC_2_-BQQDI and the thiol MeSTP for the contact functionalization in the bottom-contact TFTs. The TFTs have a channel length (*L*) of 8 μm. (The transfer and output characteristics of the TFTs having a channel length of 80 μm are shown in Figure S10.)

The current–voltage characteristics of the TFTs fabricated on flexible PEN are similar to those of the TFTs fabricated on silicon substrates. The turn-on voltage of the N1100 TFTs (about −1 V) is again somewhat more negative than that of the PhC_2_-BQQDI TFTs (−0.2 to −0.5 V). Regardless of the device architecture (top or bottom contacts), the TFTs have effective charge-carrier mobilities (*μ*_eff,lin_) of about 0.3 cm^2^ V^–1^ s^–1^ for N1100 and 0.6 cm^2^ V^–1^ s^–1^ for PhC_2_-BQQDI (for a channel length of 80 μm; Figure S10), which are only slightly smaller than those of the TFTs fabricated on silicon substrates.

The results of the TLM analysis for the TFTs fabricated on flexible PEN substrates are summarized in Figure 8 and Figures S11 and S12 and Table 5. The top-contact TFTs have contact resistances of 2.2 kΩ cm for N1100 and 4.6 kΩ cm for PhC_2_-BQQDI (slightly smaller than those in the TFTs fabricated on silicon substrates, for reasons that are not clear). The contact resistances of the bottom-contact TFTs (with MeSTP-functionalized contacts) are again notably smaller compared to the top-contact TFTs, 1 kΩ cm for N1100 and 0.21 kΩ cm for PhC_2_-BQQDI. The contact resistance of 0.21 kΩ cm is the smallest contact resistance reported to date for flexible n-channel organic TFTs (see Table 6). The intrinsic channel mobility (*μ*_0_) of the flexible TFTs reaches 0.95 cm^2^ V^–1^ s^–1^ (for the bottom-contact PhC_2_-BQQDI TFTs; see Table 5).

**Figure 8:**
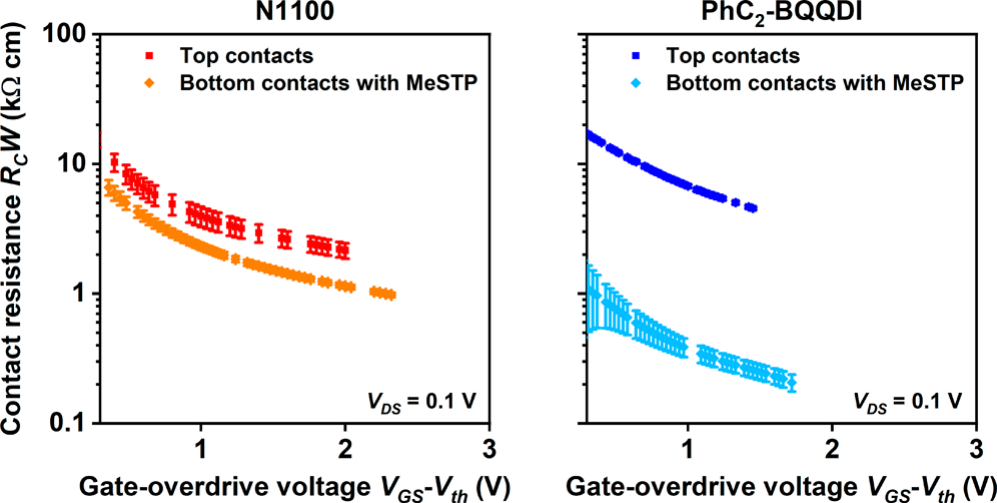
Contact resistance (*R*_*C*_ *W*) of top-contact and bottom-contact TFTs fabricated on flexible PEN substrates using N1100 or PhC_2_-BQQDI as the semiconductor and MeSTP for the contact functionalization in the bottom-contact TFTs, extracted with the transfer length method and plotted as a function of the gate-overdrive voltage (*V*_GS_-*V*_th_). The minimum contact resistances extracted from these measurements are summarized in Table 4.

**Table 5:** Intrinsic Channel Mobility (μ_0_) and Contact Resistance (*R*_*C*_ *W*) of Top-Contact and Bottom-Contact TFTs Fabricated on Flexible PEN Substrates Using N1100 and PhC_2_-BQQDI as the Semiconductor and MeSTP for the Contact Functionalization in the Bottom-Contact TFTs, Extracted with the Transfer Length Method at the Largest Gate-Overdrive Voltage (*V*_*GS*_-*V*_*th*_) in the Linear Regime of Operation (*V*_DS_ = 0.1 V; *V*_GS_ = 2 V) and Averaged over the Number of TFTs Given in Parentheses (see Figure 8 and Figures S10, S11, and S12)

semiconductor	device architecture/thiol	maximum intrinsic mobility *μ*_0_	average intrinsic mobility μ_0_	minimum contact resistance *R* *_C_* *W*	average contact resistance *R* *_C_* *W*
		(cm^2^ V^–1^ s^–1^)	(cm^2^ V^–1^ s^–1^)	(kΩ cm)	(kΩ cm)
N1100	TC/none^4^	0.89	0.64 ± 0.20	2.2	6.7 ± 5.3
	BC/MeSTP^2^	0.57	0.51 ± 0.09	1.0	2.9 ± 2.7
PhC_2_-BQQDI	TC/none^2^	0.93	0.86 ± 0.09	4.4	7.7 ± 4.4
	BC/MeSTP^11^	0.95	0.77 ± 0.09	0.21	0.49 ± 0.23

**Table 6:** Literature Summary of the Smallest Contact Resistance (*R*_*C*_ *W*) Reported Previously for n-Channel Organic TFTs on Rigid or Flexible Substrates Operated in Nitrogen or in Air, and for TFTs Based on N1100 or PhC_2_-BQQDI^a^

*R*_*C*_ *W* (kΩ cm)	semiconductor	substrate	device architecture	measured in	reference
0.3	P(NDI2OD-T2)	AlN	top-gate	nitrogen	^17^
1.2	BASF GSID104031–1	glass	top-contact	air	^18^
1.0	P(NDI2OD-T2)	flexible PEN	top-gate	nitrogen	^19^
15	N1100	flexible PEN	top-contact	air	^20^
4.7	PhC_2_-BQQDI	silicon	top-contact	air	^43^
1.9	TAPP-Br_4_	silicon	bottom-contact	air	this work
0.49	N1100	silicon	bottom-contact	air	this work
1.0	N1100	flexible PEN	bottom-contact	air	this work
0.13	PhC_2_-BQQDI	silicon	bottom-contact	air	this work
0.21	PhC_2_-BQQDI	flexible PEN	bottom-contact	air	this work

a The results reported here are shown for comparison.

In addition to a very small contact resistance, the flexible TFTs also have a large on/off current ratio (10^7^) and an excellent subthreshold swing, 92 mV decade^–1^ for N1100 and 77 mV decade^–1^ for PhC_2_-BQQDI (see Figure S15). The latter is the smallest subthreshold swing reported to date for flexible n-channel organic TFTs operated in air. (Subthreshold swings of 69 and 70 mV decade^–1^ have been reported for n-channel organic TFTs operated in vacuum and for n-channel organic TFTs fabricated on glass substrates.^58,59^ )

From simulations based on the current-crowding model and experiments performed on staggered TFTs, Jung et al. proposed a contact-resistance model to extract the power-law dependence of the intrinsic channel mobility and the contact resistance on the gate-source voltage, as well as their gate-source-voltage-independent values (*μ*_0_ and *R*_*C*_^∞^).^60,61^ They observed that the intrinsic channel mobility increases and the contact resistance decreases with an increasing gate-overdrive voltage. They attributed the increase in intrinsic channel mobility to a shift of the Fermi level toward the transport level and the decrease in contact resistance to an increase in carrier mobility and carrier density with increasing gate-overdrive voltage. Figure S14 shows how the contact resistance *R*_*C*_ *W* and the intrinsic channel mobility *μ*_0_ of our TFTs depend on the gate-overdrive voltage *V*_GS_-*V*_th_. In Figure S15, the formalism reported by Kraft et al. was employed to determine the gate-source-voltage-independent contact resistance (*R*_*C,*0_ *W*).^62^ This formalism is identical to the power-law contact resistance model proposed by Jung et al.^61^ by assuming γ = 0 and *V*_th_^eff^ = *V*_th_, which leads to *R_C,0_* *W* = *R*_*C*_^*∞*^ *W*. The first assumption (γ = 0) is justified by the observation that the intrinsic channel mobility of our TFTs is quite constant over a wide range of gate-overdrive voltages (see Figure S14). The second assumption (*V*_th_^eff^ = *V*_th_) is justified by the fact that our TLM measurements were performed with an extremely small drain-source voltage (*V_DS_* = 0.1 V). Depending on the type of semiconductor, the device architecture, and the type of thiol used for the contact functionalization, the values of *R*_*C,*0_ *W* of our TFTs range from physically meaningful (positive) values in the case of staggered N1100 TFTs to physically unrealistic *R*_*C,*0_ *W* values for the PhC_2_-BQQDI TFTs (95 % of which are negative). This leads to the conclusion that for coplanar TFTs, for which we obtained physically unrealistic negative values for the gate-source voltage-independent contact resistance *R*_*C,*0_ *W* (see Figure S15), the model developed by Jung et al. may not be applicable.

The results of bias-stress measurements performed on top- and bottom-contact PhC_2_-BQQDI TFTs with and without contact functionalization using MeSTP fabricated on PEN substrates are summarized in Figures S16 and S17.

During bias stress, gate-source and drain-source voltages of 2 V were applied continuously for 24 h. The results show that the TFTs have very similar bias-stress stability regardless of the device architecture and contact functionalization.

The long-term stability of bottom-contact PhC_2_-BQQDI TFTs using MeSTP for the contact functionalization fabricated on PEN substrates is illustrated in Figure S18. In the present study, the TFTs were measured once more 2.5 years after their fabrication. The results show that the TFTs degrade significantly over a period of 2.5 years due to a decrease in the intrinsic channel mobility and an increase in contact resistance. Since the contact resistance degrades faster than the intrinsic channel mobility, the degradation is more pronounced in TFTs with shorter channel lengths (*L* = 8 μm) where the increase in contact resistance has a larger impact.

The results of bending tests performed on bottom-contact PhC_2_-BQQDI TFTs fabricated on a flexible PEN substrate using MeSTP for the contact functionalization are summarized in Figures S19 and S20. As can be seen, the contact resistance of the TFTs increases when the TFTs are being bent and the contact resistance recovers when the TFTs are measured in the flat state after bending.

## Conclusions

Unlike the charge-carrier mobility in organic semiconductors, which can be predicted with increasingly good accuracy by computational methods,^63,64^ the contact resistance in organic TFTs is still difficult to predict from theory. To determine which combination of materials from a set of three promising organic semiconductors and four contact-functionalization thiols would provide the smallest contact resistance, we therefore conducted an empirical study. For this, we fabricated low-voltage n-channel organic TFTs based on the three vacuum-deposited small-molecule semiconductors TAPP-Br_4_, N1100, and PhC_2_-BQQDI. The TFTs were fabricated both on rigid silicon and on flexible polymeric substrates, and both in the inverted staggered (bottom-gate, top-contact) and in the inverted coplanar (bottom-gate, bottom-contact) device architecture. Gold was used as the source/drain metal, and the contacts of the bottom-contact TFTs were functionalized with a chemisorbed monolayer of methylthiophenol (MeTP), methoxythiophenol (MeOTP), methylsulfanylthiophenol (MeSTP), or benzyl mercaptan (BM) to minimize the contact resistance. The best device performance was obtained for PhC_2_-BQQDI TFTs with MeSTP-functionalized bottom contacts, for which we measured contact resistances of 130 Ω cm for TFTs fabricated on silicon substrates and 210 Ω cm for TFTs on flexible polymeric substrates; these are the smallest contact resistances reported to date for n-channel organic TFTs, despite the fact that the measurements were carried out in ambient air. The observation that functionalizing the contacts with MeSTP leads to the smallest contact resistance is consistent with the fact that MeSTP provides the lowest effective work function. In addition, the flexible bottom-contact PhC_2_-BQQDI TFTs show a subthreshold swing of 77 mV decade^–1^; this is the smallest subthreshold swing reported to date for flexible n-channel organic TFTs operated in ambient air. These results provide a significant improvement in view of the realization of low-voltage organic complementary circuits for low-power flexible electronics applications.

## Experimental Section

### Materials

2,9-bis­(Heptafluoropropyl)-4,7,11,14-tetrabromo-1,3,8,10-tetraazaperopyrene (TAPP-Br_4_) was synthesized as reported previously.^27^ *N,N*′-bis­(2,2,3,3,4,4,4-fluorobutyl)-(1,7 and 1,6)-dicyano-perylene-tetracarboxylic diimide (ActivInk N1100) was procured from Polyera Corp. (Skokie, IL, U.S.A.), diphenylethyl-3,4,9,10-benzo­[de]­isoquinolino­[1,8-gh]­quinolinetetracarboxylic diimide (PhC_2_-BQQDI) from Fujifilm Wako Pure Chemical Cooperation (Neuss, Germany), *n*-tetradecylphosphonic acid from PCI Synthesis (Newburyport, MA, U.S.A.), 4-methylbenzenethiol (MeTP) and benzyl mercaptan (BM) from Sigma-Aldrich, 4-methoxythiophenol (MeOTP) and 4-(methylsulfanyl)-thiophenol (MeSTP) from TCI Deutschland GmbH (Eschborn, Germany), and 125 μm-thick polyethylene naphthalate (PEN) substrates from Inabata Europe GmbH (Düsseldorf, Germany). For the *in situ* spectroelectrochemical measurements, float-glass substrates coated with a layer of indium tin oxide (ITO; electrical sheet resistance <20 Ω sq^–1^) were procured from Präzisions Glas & Optik GmbH (PGO; Iserlohn, Germany).

### Device Fabrication

TFTs were fabricated on heavily doped silicon or flexible PEN substrates, either in the inverted staggered (bottom-gate, top-contact) or inverted coplanar (bottom-gate, bottom-contact) device architecture. For all TFTs, aluminum with a thickness of 25 nm was deposited as the gate electrode by thermal evaporation in vacuum.^65^ The film thickness of the vacuum-deposited films was monitored by using a quartz crystal microbalance. For the TFTs on silicon, the gate electrodes were not patterned and thus cover the entire substrate, while for the TFTs on PEN, a silicon stencil mask was used to obtain patterned gate electrodes.^66^ The aluminum surface was exposed to oxygen plasma to form a thin aluminum oxide (AlO_*x*_) layer, followed by immersing the substrates into a 2-propanol solution of *n*-tetradecylphosphonic acid for 3 to 4 h to form a self-assembled monolayer (SAM) on the AlO_x_ surface.

This results in a hybrid AlO_*x*_/SAM gate dielectric with a total thickness of about 8 nm and a unit-area capacitance (*C*_diel_) of 0.6 μF cm^–2^.^67^

For the top-contact TFTs, the next process step is the deposition of the organic-semiconductor layer, followed by the deposition of the source and drain contacts. The organic-semiconductor layer was deposited by thermal sublimation in vacuum (on silicon substrates without a mask; on PEN through a stencil mask). For the top-contact TFTs, the semiconductor has a nominal thickness of 20 nm. During the semiconductor deposition, the substrate was held at a temperature of 90 °C for TAPP-Br_4_ and at a temperature of 140 °C for N1100 and PhC_2_-BQQDI. The gold source and drain contacts were deposited by thermal evaporation in vacuum, with a thickness of 30 nm and patterned using a stencil mask. For the bottom-contact TFTs, the source and drain contacts were deposited prior to the organic semiconductor.

After the deposition of the Au source and drain contacts, the substrates were immersed into a 10 mM ethanol solution of either MeTP, MeOTP, MeSTP, or BM for 5 h to form a chemisorbed monolayer on the Au surface to reduce the contact resistance. The last process step is the deposition of the organic-semiconductor layer, which has a nominal thickness of 30 nm for the bottom-contact TFTs. The TFTs have channel lengths ranging from 2 to 80 μm and a channel width of 200 μm. Bias-stress measurements and bending tests were performed on TFTs fabricated on PEN substrates.

### TFT Characterization

The gate-dielectric capacitance was measured by using a Hameg HM8118 LCR Meter. The current–voltage characteristics of the TFTs were measured using an Agilent 4156C Precision Semiconductor Parameter Analyzer, controlled using the software “SweepMe!” (https://sweep-me.net). All electrical measurements were performed in ambient air at room temperature (20 °C, relative humidity 30–60 %) under yellow laboratory light.

During the bias-stress measurements, gate-source and drain-source voltages of 2 V were continuously applied for 24 h, and the transfer characteristics were measured before and after bias stress.

### Experimental Estimate of the LUMO Energies of the Organic Semiconductors

*In situ* spectroelectrochemical measurements on the organic-semiconductor films were performed using an Autolab PGSTAT101 potentiostat (Metrohm) and a Zeiss UV–vis spectrometer equipped with an MCS621 Vis II spectrometer cassette and a CLH600F lamp in a custom-built three-electrode, one-compartment quartz cell at room temperature under an argon atmosphere.^51,68^ The electrolyte is a solution of 0.1 M tetrabutylammonium hexafluorophosphate salt (TBAPF_6_) in acetonitrile (for TAPP-Br_4_ and PhC_2_-BQQDI) or dichloromethane (for N1100), deaerated by argon bubbling. The pseudoreference electrode (AgCl coated Ag wire) and the counter electrode (Pt wire) were immersed directly into the electrolyte solution. For measurements on vacuum-deposited thin-films of TAPP-Br_4_ and PhC_2_-BQQDI, the working electrode was a float-glass substrate coated with a layer of ITO onto which a 30 nm-thick film of the organic semiconductor was deposited by thermal sublimation in vacuum. Background absorption measurements were performed using an ITO-coated float-glass substrate without an organic-semiconductor layer to serve as a reference during the absorption measurements performed under the same conditions.^69^ The absorption spectra of the vacuum-deposited organic-semiconductor films were recorded in transmission mode. Measurements on vacuum-deposited thin-films of N1100 were unsuccessful since the N1100 films were dissolved from the working electrode upon immersion into the electrolyte. Electrochemical measurements on N1100 were thus performed by dissolving 0.6 mM of N1100 in a dichloromethane/TBAPF_6_ electrolyte. For N1100, the measurements were performed under thin-layer conditions, which ensures complete reduction of the molecules and a direct spectroscopic identification. The three-electrode setup allows for measurements in reflection mode using a polished platinum disc sealed in glass as a mirror-type working electrode.^68,70^ All *in situ* spectroelectrochemical measurements were performed with a scan rate of 0.02 V s^–1^ and a potential step of 0.005 V, i.e., the data points of the electrochemical experiment were recorded with a time interval of 0.25 s. The spectrometer was set to record a new spectrum with a time interval of 0.25 s as well.

The potentials were rescaled to the formal potential of the redox couple ferrocene/ferrocenium (Fc/Fc^+^) (external standard). The half-wave potentials were calculated from the cyclic-voltammetry (CV) curves as follows:

$$E_{1/2}=(E_{p,\mathrm{red}}+E_{p,\mathrm{ox}})/2$$ 7

where *E*_*p,*red_ and *E*_*p,*ox_ are the peak potentials of the reduction and the oxidation. The LUMO energies were calculated assuming a formal potential of −4.8 eV for the ferrocene/ferrocenium redox couple (Fc/Fc^+^) on the Fermi scale,^53^ according to the following equation:

$$E_{\mathrm{LUMO}}=-(E_{o\mathrm{nset}}+4.8)\;[\mathrm{eV}]$$ 8

## Supplementary Material



## Notes

Output characteristics of top-contact TFTs and bottom-contact PhC_2_-BQQDI TFTs fabricated on silicon substrates, measured unit-area capacitance of the AlO_*x*_/SAM dielectric, SEM images of TFT channels fabricated on silicon and flexible PEN substrates, summary of important device parameters (effective mobility, threshold voltages...) for TFTs fabricated on silicon and flexible PEN substrates, TLM analysis for all combinations of semiconductor-thiol bottom-contact TFTs on silicon substrates and for top-contact and bottom-contact TFTs fabricated on flexible PEN substrates, transfer and output characteristics for N1100 and PhC_2_-BQQDI TFTs on flexible substrates (*L* = 80 μm), transfer characteristics of the TFTs with the lowest subthreshold swing, bias-stress measurements on top-contact and bottom-contact TFTs without and with MeSTP for contact functionalization, bending test on TFTs fabricated on PEN substrates, details on the DFT calculations including xyz-coordinates for the optimized structures of TAPP-Br_4_, N1100 and PhC_2_-BQQDI (PDF)
