# Supplementary material for: Flexible n‑Channel Organic Transistors with Low Contact Resistance
Source: ACS Appl Mater Interfaces. 2025 Dec 22;18(1):2162–75. doi: 10.1021/acsami.5c17364 (PMC12781057; doi:10.1021/acsami.5c17364)
Supplement: Supplementary file 1 [file am5c17364_si_001.pdf]

Supporting Information

Flexible n-Channel Organic Transistors with Low Contact Resistance

Sabrina Steffens[a], Tobias Wollandt[a], Karla Cordero-Solano[a], Robert Eichelmann[b], Alexander Kochan[b], Xiuming Sun[c], Florian Letzkus[d], Joachim N. Burghartz[d], Sabine Ludwigs[c], Lutz H. Gade[b], and Hagen Klauk[a]*

[a] Max Planck Institute for Solid State Research, Heisenbergstraße 1, 70569 Stuttgart, Germany

** E-mail: S.Steffens@fkf.mpg.de*

[b] Anorganisch-Chemisches Institut, Universität Heidelberg, Im Neuenheimer Feld 270, 69120 Heidelberg, Germany

[c] IPOC - Functional Polymers, Institute of Polymer Chemistry (IPOC), Universität Stuttgart, Pfaffenwaldring 55, 70569 Stuttgart, Germany

[d] Institut für Mikroelektronik Stuttgart (IMS CHIPS), Allmandring 30a, 70569 Stuttgart, Germany

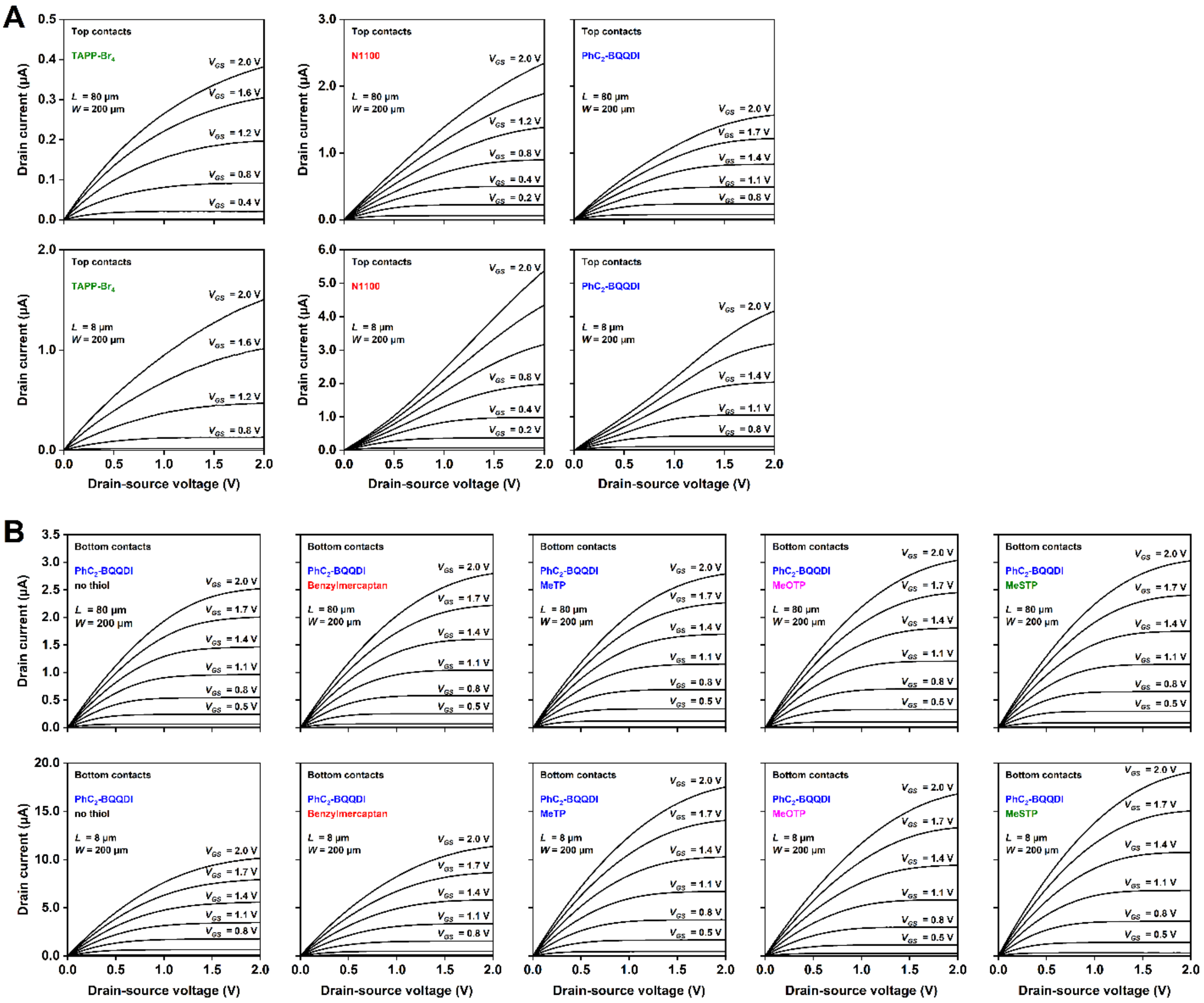

Figure S1. A) Output characteristics of top-contact TFTs fabricated on silicon substrates using all three semiconductors (TAPP-Br$_4$, N1100, PhC$_2$-BQQDI). The TFTs have a channel length (L) of 80 µm (first row) or 8 µm (second row). B) Output characteristics of bottom-contact TFTs fabricated on silicon substrates using PhC$_2$-BQQDI as semiconductor and either no thiol, BM, MeTP, MeOTP or MeSTP for the contact functionalization. The TFTs have a channel length (L) of 80 µm (third row) or 8 µm (fourth row).

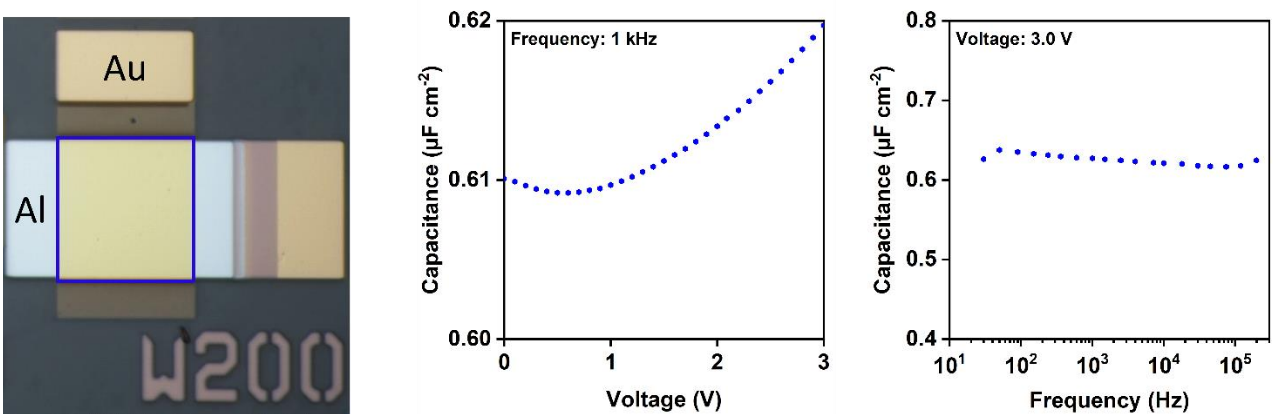

Figure S2. Left: Photograph of an Al/AlO_x/SAM/Au capacitor, fabricated for the purpose of measuring the gate-dielectric capacitance. The Al bottom and Au top electrodes (patterned by stencil lithography) define a capacitor area of 200 µm x 200 µm. Center and right: Measured unit-area capacitance of the AlO_x/SAM gate dielectric plotted as a function of the measurement frequency and as a function of the applied voltage.

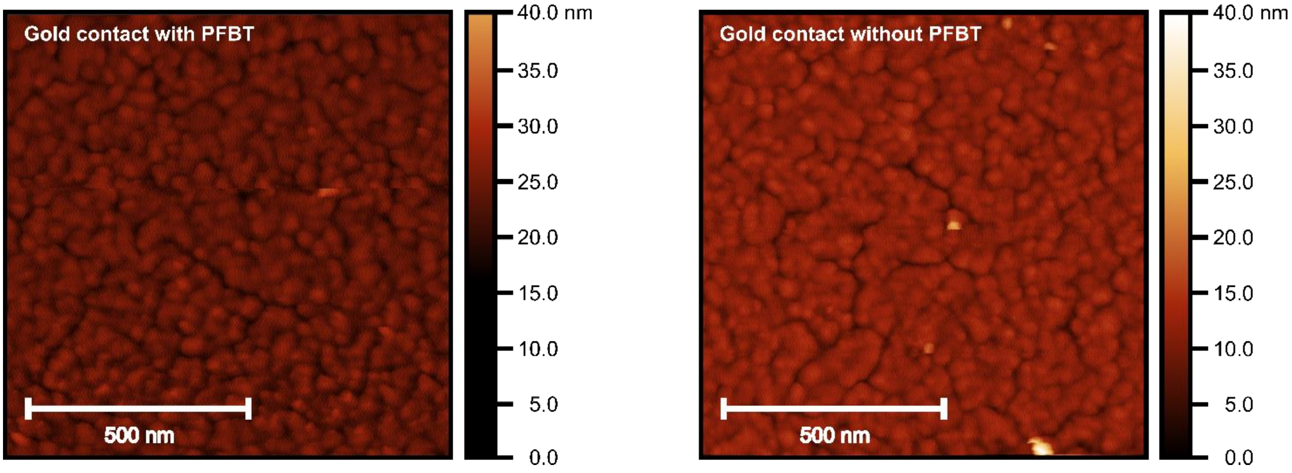

Figure S3. Atomic force microscopy (AFM) images of a PFBT-functionalized gold contact (left) and a gold contact without thiol functionalization (right). The root-mean square surface roughness is 1.90 nm with PFBT and 1.99 nm without PFBT, indicating that there is no significant difference in surface roughness.

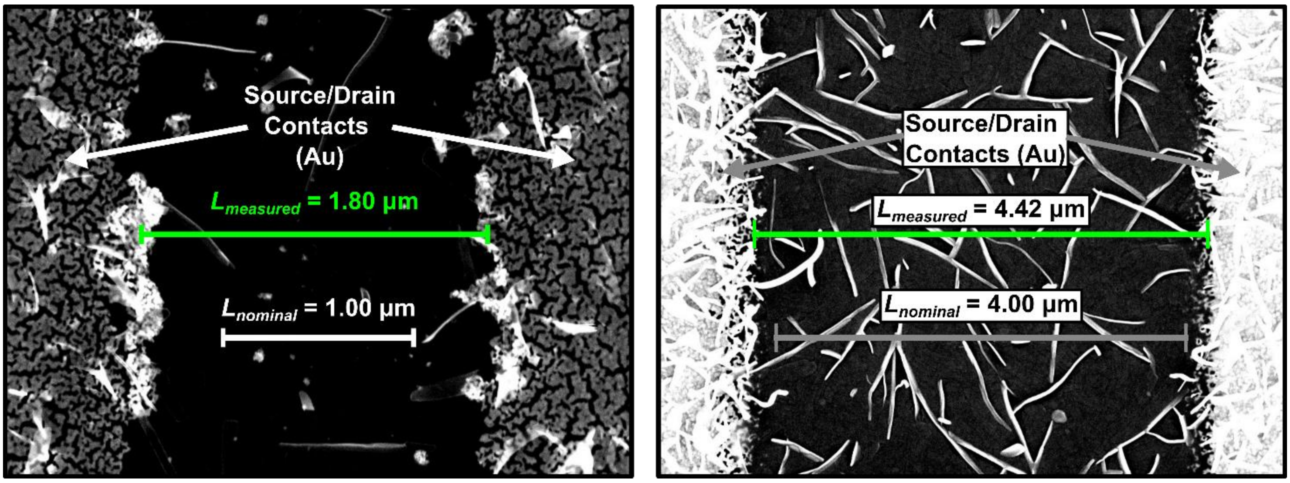

Figure S4. Left: SEM image of a bottom-contact TFT fabricated on a silicon substrate with a nominal channel length of 1 µm. Right: SEM image of a bottom-contact TFT fabricated on a flexible PEN substrate with a nominal channel length of 4 µm. The SEM images indicate that the actual channel lengths of the TFTs are larger than the nominal channel lengths by 0.4 and 0.8 µm.

Table S1. Effective charge-carrier mobilities ($\mu_{eff,lin}$ and $\mu_{eff,sat}$) and threshold voltages (V_{th}) of top-contact and bottom-contact TFTs fabricated on silicon substrates using all three semiconductors and all four thiols for two representative channel lengths (80 µm, 8 µm), extracted from the measured transfer characteristics and averaged over the number of TFTs given in parentheses.

Semiconductor	Device architecture/thiol	Channel length L (µm)	Linear effective mobility $\mu_{eff,lin}$ ($cm^2V^{-1}s^{-1}$)	Saturation effective mobility $\mu_{eff,sat}$ ($cm^2V^{-1}s^{-1}$)	Threshold voltage V_{th} (V)
TAPP-Br_4	TC/none (4)	80	0.18 ± 0.05	0.22 ± 0.11 (5)	0.68 ± 0.28
	(3)	8	0.07 ± 0.02	0.11 ± 0.02 (2)	0.88 ± 0.07
	BC / none (5)	80	0.09 ± 0.05	0.11 ± 0.02 (2)	0.94 ± 0.24
	(5)	8	0.03 ± 0.01	0.03 (1)	1.1 ± 0.3
	BC / BM (5)	80	0.13 ± 0.12	0.08 ± 0.08 (2)	1.0 ± 0.6
	(5)	8	0.04 ± 0.04	0.05 ± 0.06 (2)	1.3 ± 0.4
	BC / MeTP (6)	80	0.13 ± 0.08	0.14 ± 0.02 (3)	0.90 ± 0.53
	(5)	8	0.07 ± 0.05	0.06 ± 0.03 (3)	1.1 ± 0.6
	BC / MeOTP (7)	80	0.12 ± 0.07	0.14 ± 0.05 (5)	0.82 ± 0.45
	(7)	8	0.07 ± 0.04	0.10 ± 0.03 (5)	1.1 ± 0.4
	BC / MeSTP (6)	80	0.14 ± 0.08	0.13 ± 0.02 (3)	0.81 ± 0.65
	(4)	8	0.07 ± 0.06	0.11 ± 0.04 (2)	0.85 ± 0.48
N1100	TC/none (10)	80	0.29 ± 0.15	0.31 ± 0.17 (8)	0.08 ± 1.05
	(9)	8	0.04 ± 0.03	0.10 ± 0.04 (6)	-0.05 ± 1.05
	BC / none (5)	80	0.43 ± 0.21	0.33 ± 0.25 (2)	-0.49 ± 0.18
	(5)	8	0.15 ± 0.09	0.15 ± 0.11 (2)	-0.39 ± 0.39
	BC / BM (6)	80	0.49 ± 0.13	0.36 ± 0.07 (2)	-0.17 ± 0.50
	(6)	8	0.16 ± 0.07	0.20 ± 0.02 (2)	0.11 ± 0.64
	BC / MeTP (7)	80	0.47 ± 0.20	0.26 ± 0.02 (3)	-0.33 ± 0.38
	(7)	8	0.24 ± 0.12	0.13 ± 0.04 (3)	-0.51 ± 0.42
	BC / MeOTP (8)	80	0.49 ± 0.21	0.32 ± 0.14 (6)	-0.15 ± 0.57
	(9)	8	0.24 ± 0.12	0.19 ± 0.09 (6)	-0.04 ± 0.62
	BC / MeSTP (10)	80	0.52 ± 0.14	0.36 ± 0.10 (6)	-0.45 ± 0.26
	(13)	8	0.27 ± 0.10	0.19 ± 0.07 (7)	-0.33 ± 0.23
PhC_2-BQQDI	TC/none (4)	80	0.57 ± 0.12	0.69 ± 0.14 (3)	0.39 ± 0.11
	(4)	8	0.10 ± 0.03	0.20 ± 0.07 (3)	0.62 ± 0.10
	BC / none (14)	80	0.65 ± 0.28	0.73 ± 0.29 (6)	0.66 ± 0.37
	(15)	8	0.25 ± 0.16	0.39 ± 0.12 (6)	0.79 ± 0.31
	BC / BM (11)	80	0.84 ± 0.15	0.81 ± 0.10 (6)	0.43 ± 0.25
	(11)	8	0.47 ± 0.13	0.62 ± 0.14 (6)	0.80 ± 0.23
	BC / MeTP (8)	80	0.86 ± 0.19	0.75 ± 0.11 (3)	0.32 ± 0.32
	(8)	8	0.48 ± 0.16	0.49 ± 0.16 (3)	0.61 ± 0.38
	BC / MeOTP (10)	80	0.80 ± 0.18	0.79 ± 0.16 (5)	0.38 ± 0.28
	(12)	8	0.46 ± 0.10	0.52 ± 0.11 (5)	0.62 ± 0.27
	BC / MeSTP (26)	80	0.74 ± 0.21	0.74 ± 0.19 (14)	0.36 ± 0.26
	(44)	8	0.44 ± 0.16	0.50 ± 0.17 (17)	0.46 ± 0.25

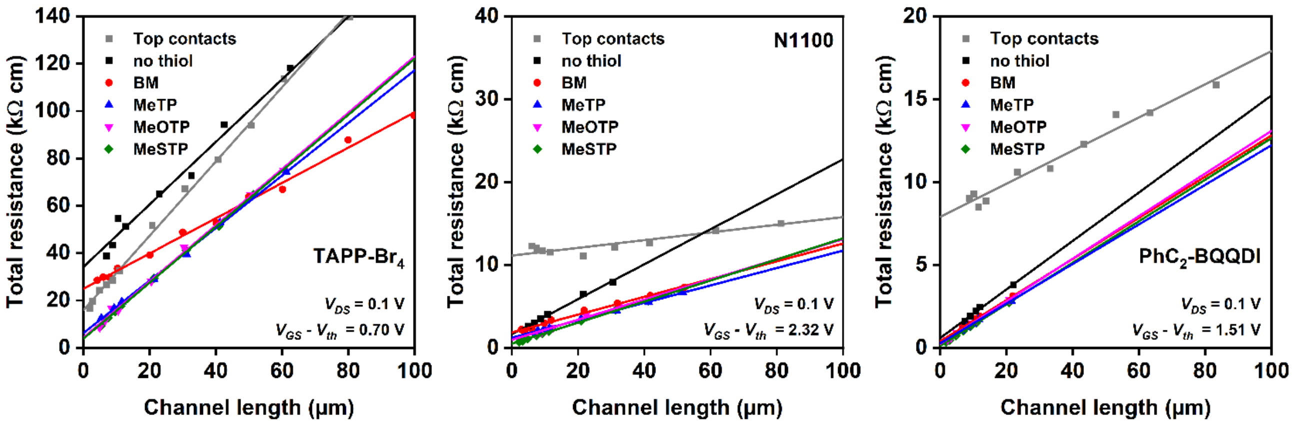

Figure S5. TLM analysis performed on top-contact and bottom-contact TFTs fabricated on silicon substrates using all three semiconductors (TAPP-Br$_4$, N1100, PhC$_2$-BQQDI) and either MeTP, MeOTP, MeSTP or BM for the contact functionalization in the bottom-contact TFTs.

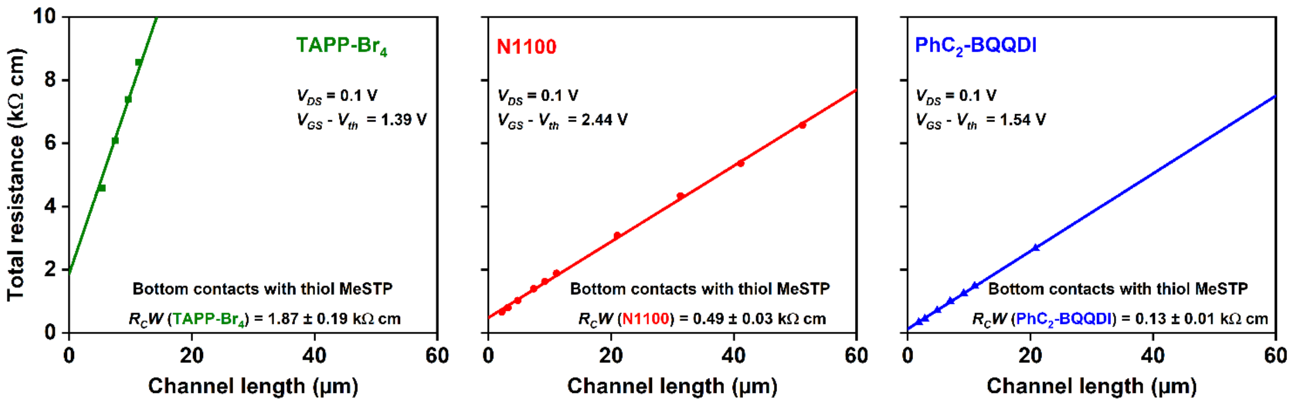

Figure S6. TLM analysis performed on bottom-contact TFTs fabricated on silicon substrates using all three semiconductors (TAPP-Br$_4$, N1100, PhC$_2$-BQQDI) and the best-performing thiol (MeSTP) for the contact functionalization.

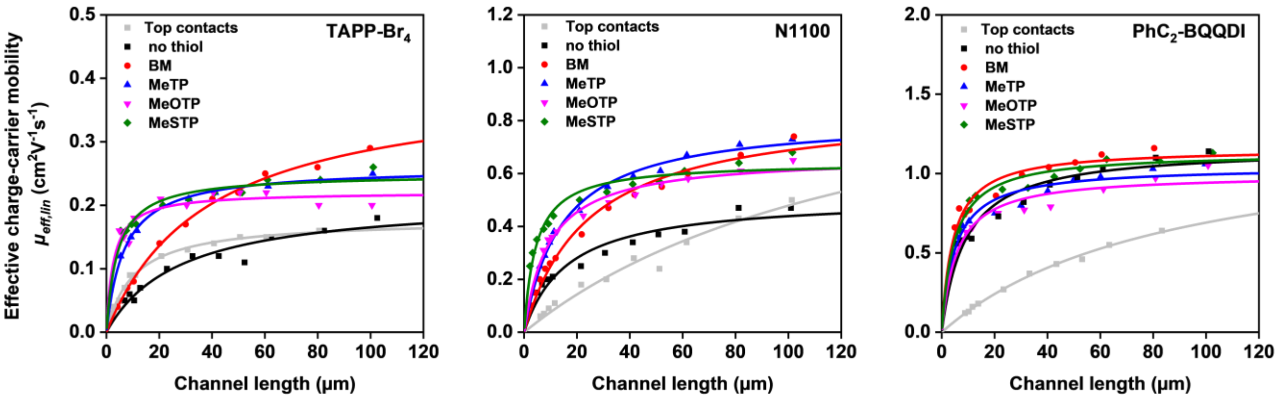

Figure S7. Effective charge-carrier mobility in the linear regime ($\mu_{eff,lin}$) extracted from the transfer characteristics of top-contact and bottom-contact TFTs fabricated on silicon substrates using all three semiconductors (TAPP-Br_4, N1100, PhC_2-BQQDI) and either MeTP, MeOTP, MeSTP or BM for the contact functionalization in the bottom-contact TFTs, plotted versus the channel length. The fit lines were calculated using Equation (6).

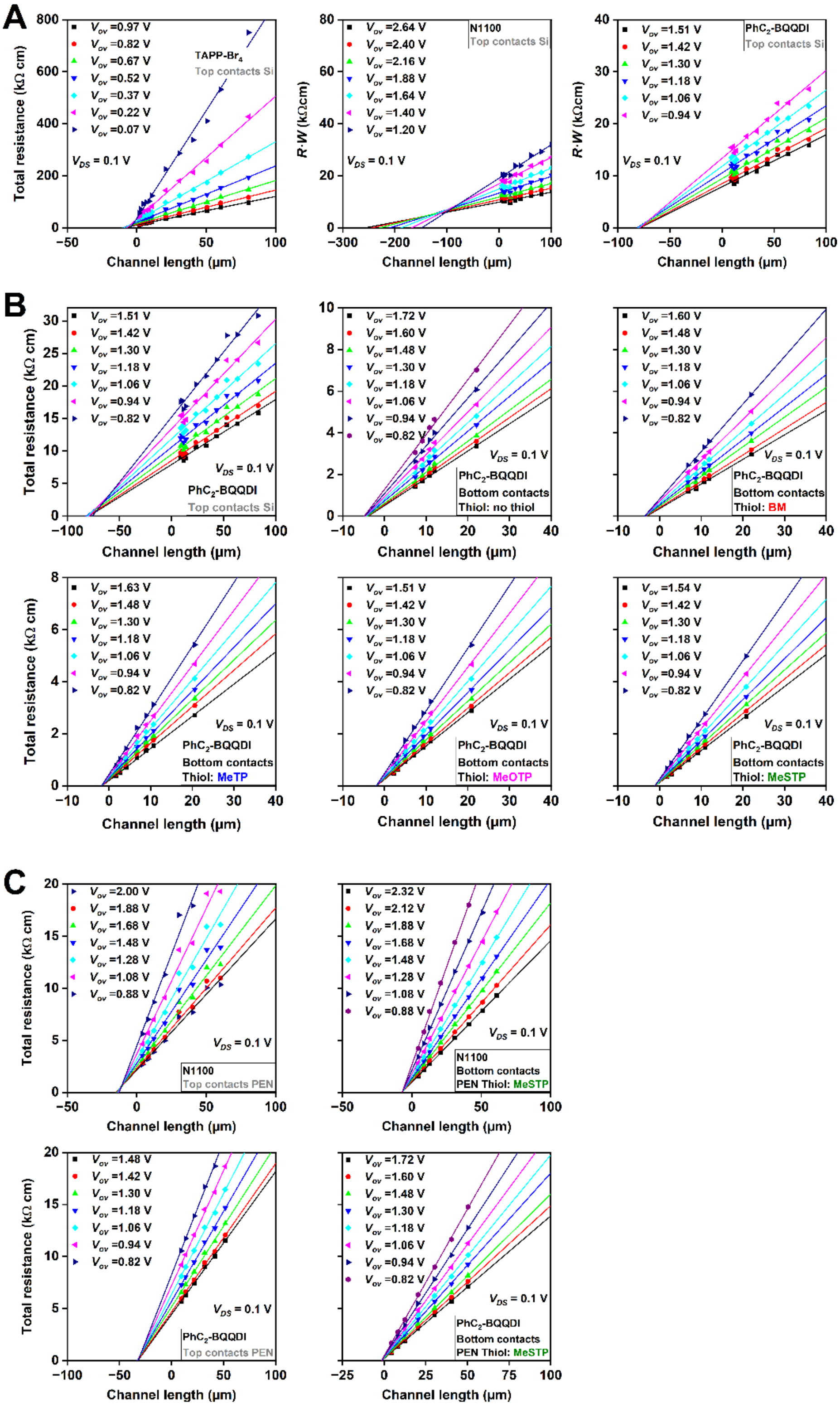

Figure S8. A) TLM analysis of top-contact TFTs fabricated on silicon substrates using all three semiconductors (TAPP-Br_4, N1100, PhC_2-BQQDI). B) TLM analysis of bottom-contact PhC_2-BQQDI TFTs fabricated on silicon substrates using either BM, MeTP, MeOTP or MeSTP for the contact functionalization. C) TLM analysis of top-contact and bottom-contact TFTs fabricated on PEN substrates using PhC_2-BQQDI or N1100 as semiconductor and MeSTP for the contact functionalization.

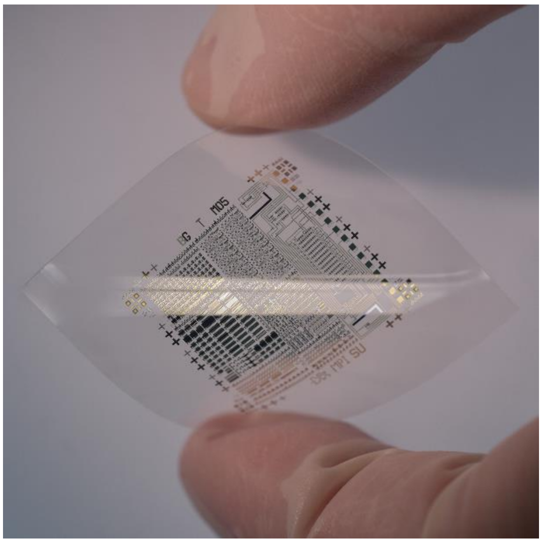

Figure S9. Photograph of TFTs fabricated on a flexible PEN substrate.

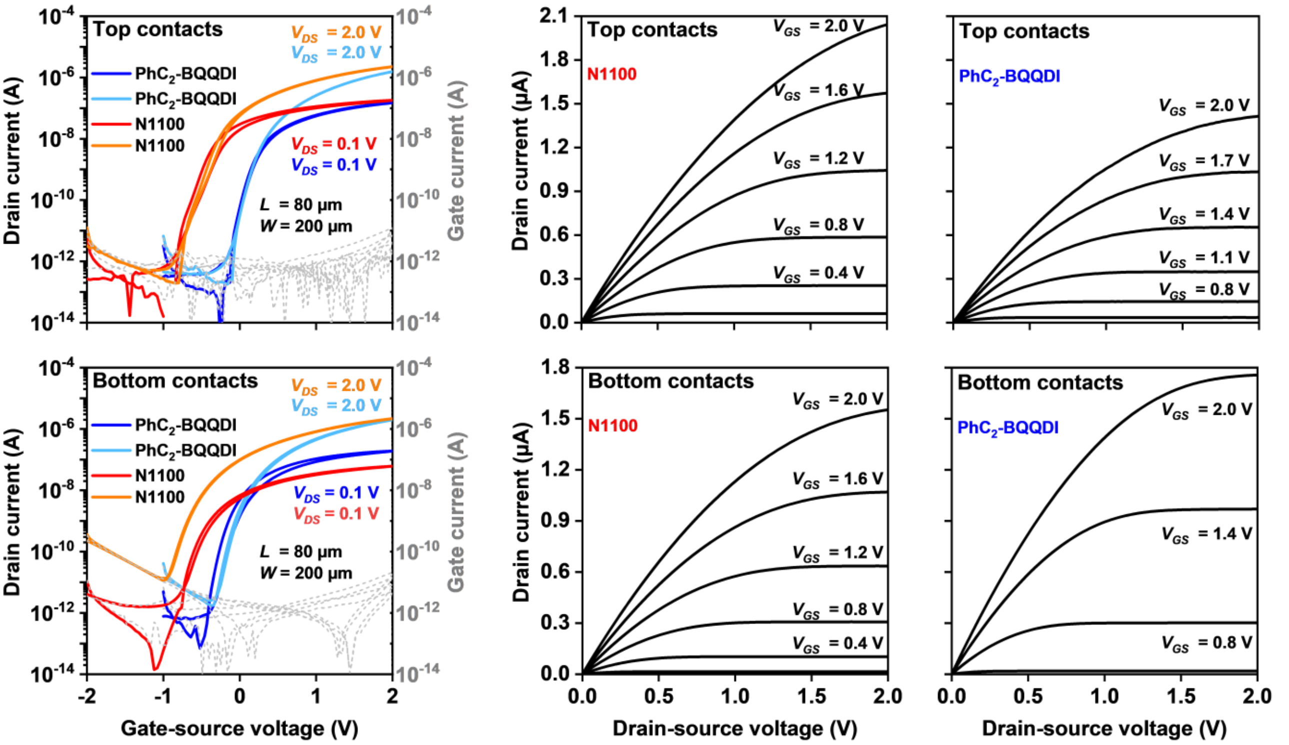

Figure S10. Transfer and output characteristics of top-contact (top row) and bottom-contact TFTs (bottom row) fabricated on flexible PEN substrates using the semiconductors N1100 and PhC_2-BQQDI and the thiol MeSTP for the contact functionalization in the bottom-contact TFTs. The TFTs have a channel length (L) of 80 µm.

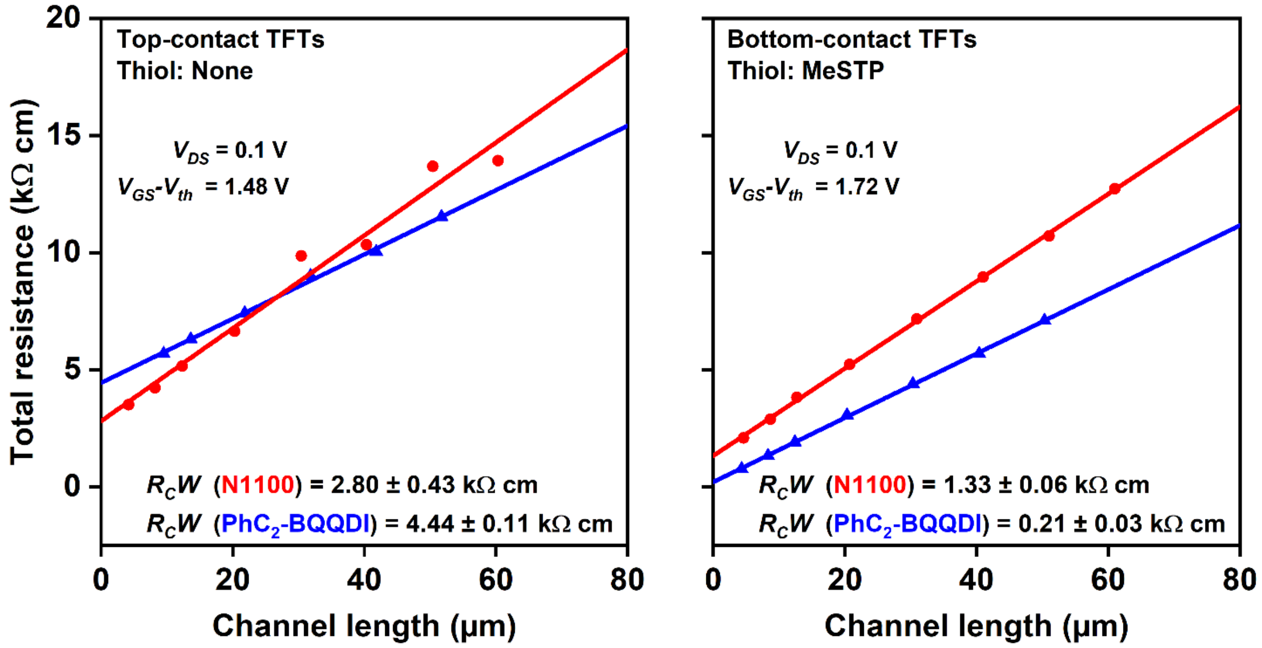

Figure S11. Results from TLM measurements performed on top-contact and bottom-contact TFTs fabricated on flexible PEN substrates using N1100 and PhC$_2$-BQQDI as semiconductors and the thiol MeSTP for contact functionalization.

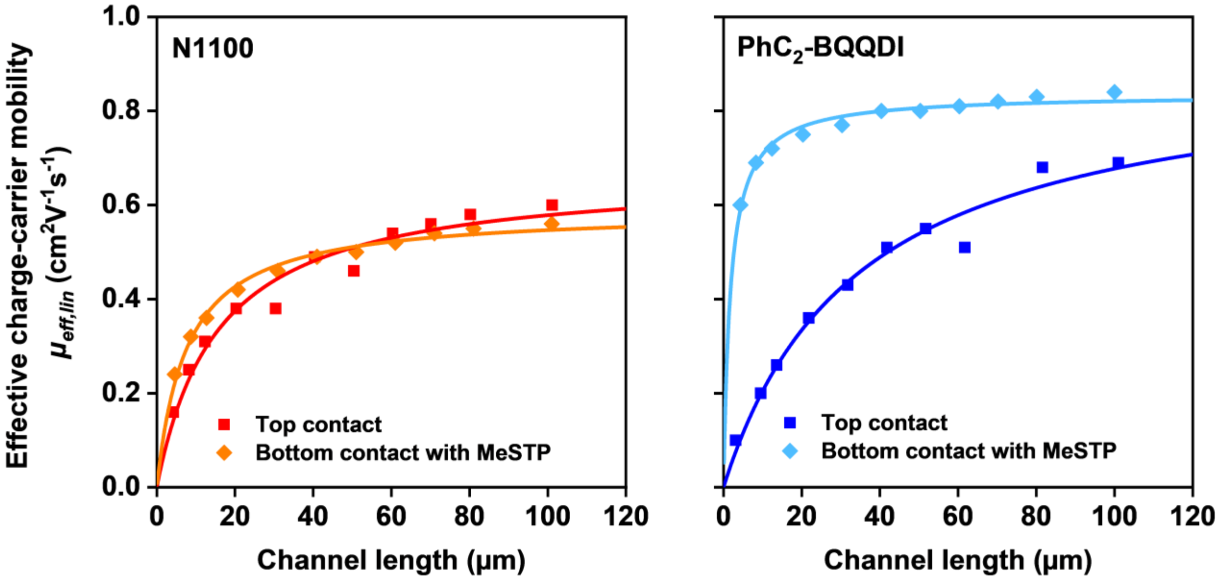

Figure S12. Effective charge-carrier mobility in the linear regime ($\mu_{eff,lin}$) extracted from the transfer characteristics of top-contact and bottom-contact TFTs fabricated on flexible PEN substrates using the semiconductors N1100 and PhC$_2$-BQQDI and the thiol MeSTP for the contact functionalization in the bottom-contact TFTs, plotted versus the channel length. The fit lines were calculated using Equation (6).

Table S2. Effective field-effect mobility, threshold voltage, subthreshold swing and on/off current ratio of top-contact and bottom-contact TFTs fabricated on flexible PEN substrates using the semiconductors N1100 and PhC_2-BQQDI and the thiol MeSTP for the contact functionalization in the bottom-contact TFTs for two representative channel lengths (80 µm, 8 µm).

Semiconductor	Device architecture / thiol	Channel length L (µm)	Linear Effective mobility $\mu_{eff,lin}$ ($cm^2V^{-1}s^{-1}$)	Saturation Effective mobility $\mu_{eff,sat}$ ($cm^2V^{-1}s^{-1}$)	Threshold voltage V_{th} (V)	Subthreshold swing (mVdecade^{-1})	On/off current ratio
N1100	TC/ none (8)	80	0.30 ± 0.15	0.30 ± 0.12	-0.42 ± 0.09	130 ± 20	5.4 ± 0.5
	(7)	8	0.12 ± 0.09	0.17 ± 0.09	-0.34 ± 0.27	126 ± 20	6.2 ± 0.5
	BC/ MeSTP (4)	80	0.31 ± 0.16	0.27 ± 0.13	-0.08 ± 0.23	141 ± 9	4.8 ± 0.3
	(3)	8	0.17 ± 0.11	0.15 ± 0.09	-0.04 ± 0.33	118 ± 19	5.6 ± 0.5
PhC2-BQQDI	TC/ none (6)	80	0.58 ± 0.11	0.68 ± 0.09	0.57 ± 0.17	105 ± 7	5.5 ± 0.4
	(6)	8	0.12 ± 0.07	0.27 ± 0.04	0.67 ± 0.32	100 ± 14	5.7 ± 0.5
	BC/ MeSTP (25)	80	0.64 ± 0.26	0.64 ± 0.24	0.40 ± 0.20	111 ± 11	5.1 ± 0.7
	(27)	8	0.53 ± 0.18	0.60 ± 0.14	0.56 ± 0.23	99 ± 19	6.1 ± 1.3

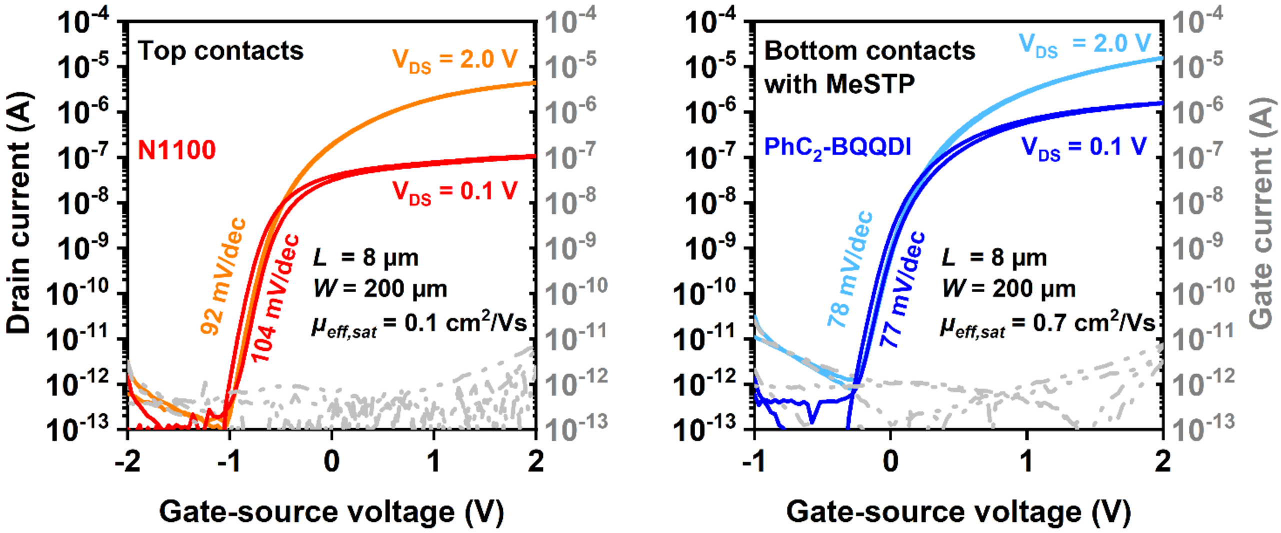

Figure S13. Transfer characteristics of a top-contact N1100 TFT (left) and a bottom-contact PhC_2-BQQDI TFT (right), both fabricated on flexible PEN substrates, using the thiol MeSTP for the contact functionalization in the bottom-contact PhC_2-BQQDI TFT. The TFTs have a channel length of 8 µm. These are the TFTs with the smallest subthreshold swings obtained in this work: 92 mV decade^{-1} for the top-contact N1100 TFT, and 77 mV decade^{-1} for the bottom-contact PhC_2-BQQDI TFT. The latter is the smallest subthreshold swing reported to date for flexible n-channel organic TFTs operated in ambient air.

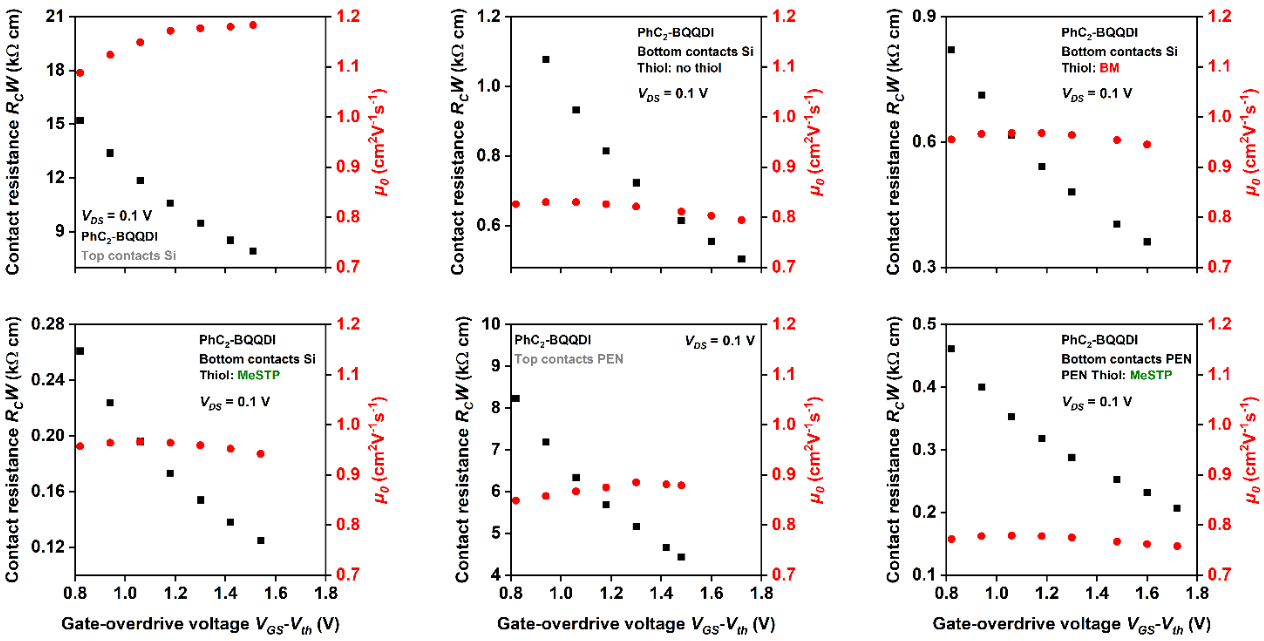

Figure S14. Channel-width-normalized contact resistance R_CW and intrinsic channel mobility μ_0 of top-contact and bottom-contact PhC$_2$-BQQDI TFTs fabricated on silicon or PEN substrates using either BM or MeSTP for the contact functionalization, plotted as a function of the gate-overdrive voltage V_{GS}-V_{th}.

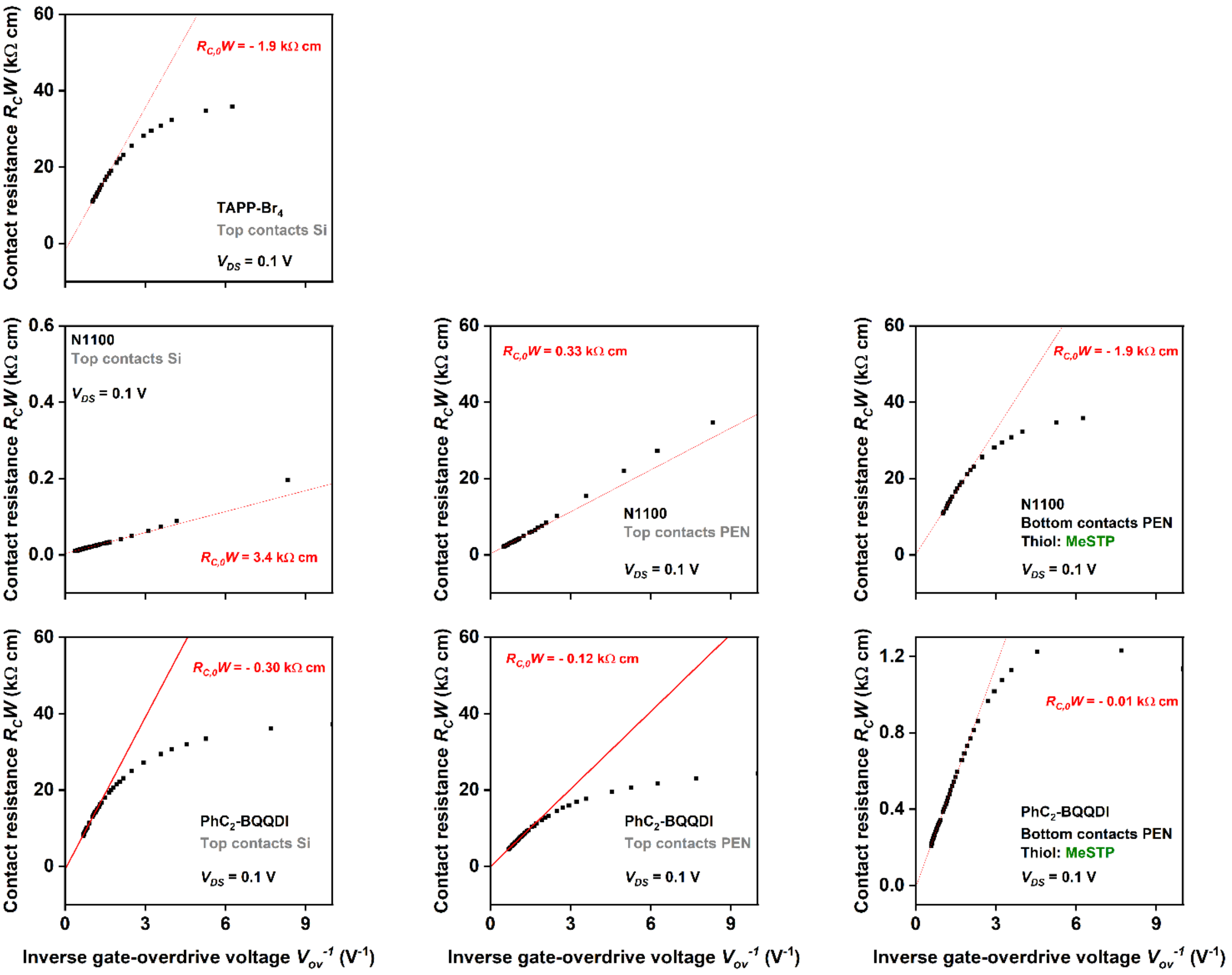

Figure S15. Channel-width-normalized contact resistance R_CW of top-contact and bottom-contact TFTs fabricated on silicon or PEN substrates using all three semiconductors (TAPP-Br_4, N1100, PhC$_2$-BQQDI) and MeSTP for the contact functionalization, plotted versus the inverse of the gate-overdrive voltage (V_{GS}-V_{th}) to extract the value of the gate-source-voltage-independent part of the contact resistance ($R_{C,0}W$).

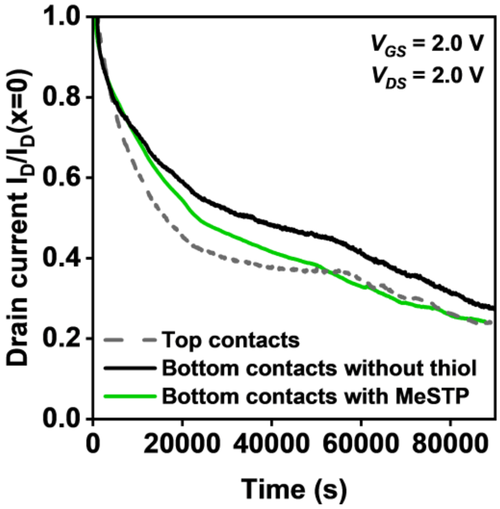

Figure S16. Bias-stress-induced decay of the drain current of top-contact and bottom-contact PhC_2-BQQDI TFTs with and without contact functionalization using MeSTP fabricated on flexible PEN substrates. Gate-source and drain-source voltages of 2 V were applied continuously for 24 hours.

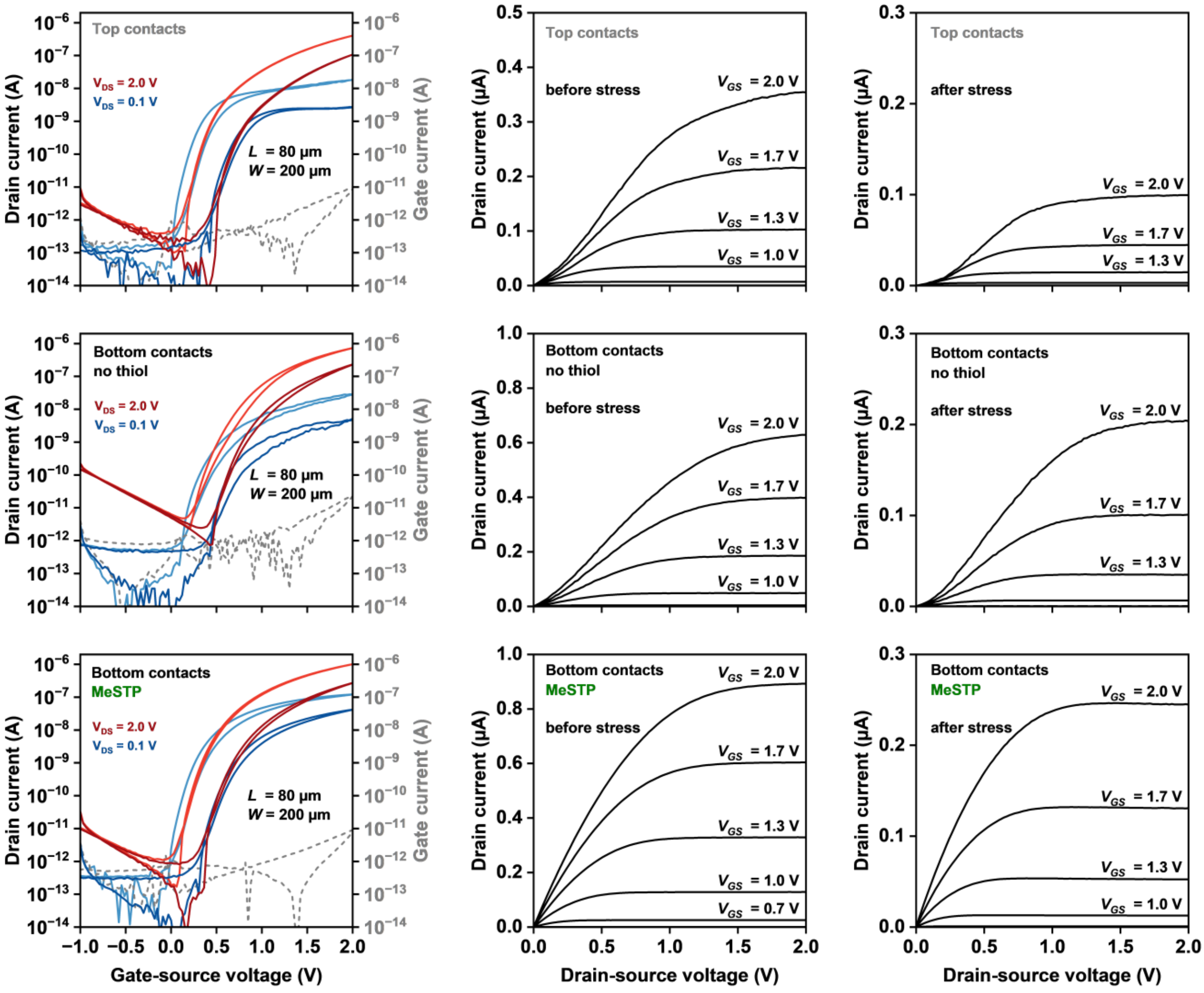

Figure S17. Transfer and output characteristics of top-contact and bottom-contact PhC_2-BQQDI TFTs fabricated on flexible PEN substrates prior to and after bias stress. The transfer characteristics were measured with drain-source voltages of 2.0 V (red) and 0.1 V (blue). During bias stress, gate-source and drain-source voltages of 2 V were applied continuously for 24 hours. The transfer characteristics measured after bias stress are shown in dark blue and dark red.

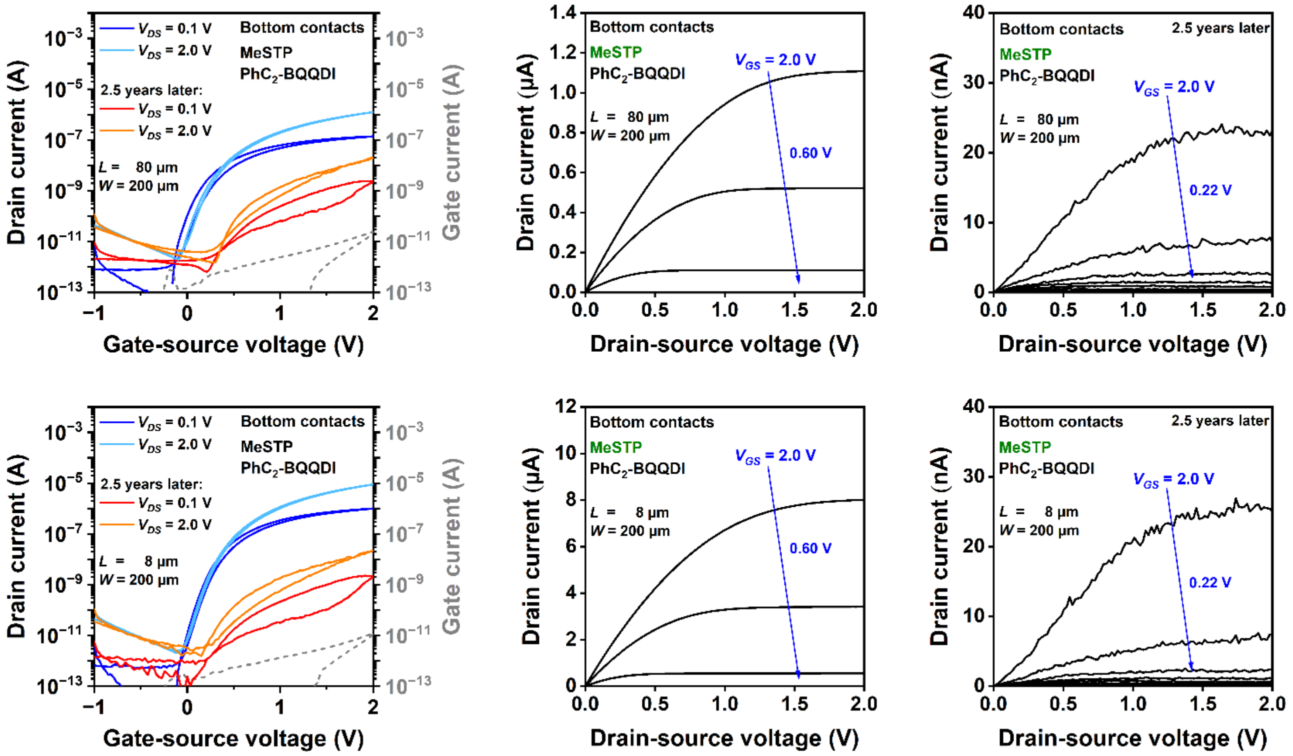

Figure S18. Transfer characteristics and output characteristics of bottom-contact PhC_2-BQQDI TFTs with MeSTP-functionalized gold contacts measured immediately after fabrication and 2.5 years after fabrication stored under ambient conditions (20°C, relative humidity between 30 and 70 %).

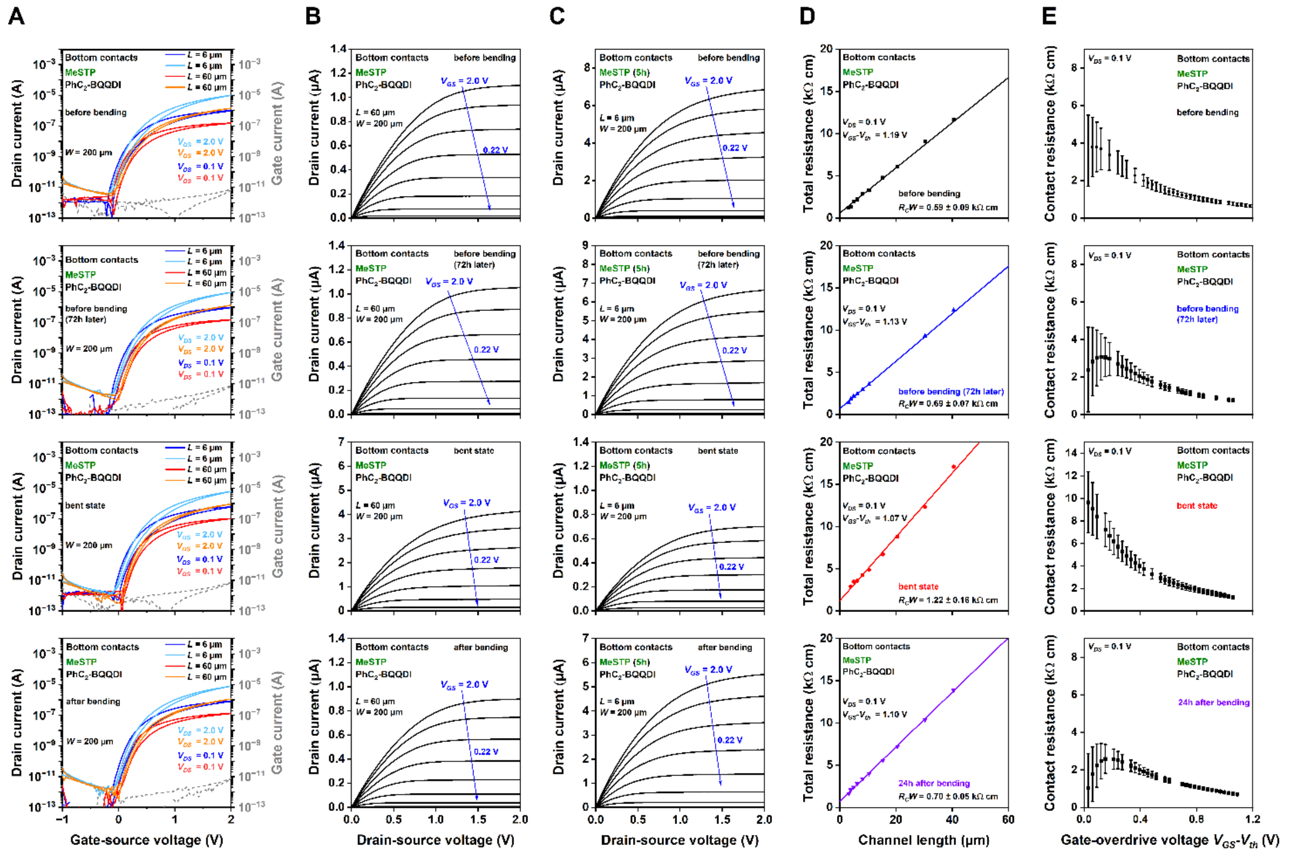

Figure S19. Bending stability of bottom-contact PhC_2-BQQDI TFTs fabricated on a flexible PEN substrate using MeSTP for the contact functionalization. A) Transfer characteristics of two TFTs ($L = 6$ µm and $L = 60$ µm) before bending, 72 h later (but still before bending), in the bent state, and after bending. B) Output characteristics of one of the TFTs ($L = 60$ µm). C) Output characteristics of the other TFT ($L = 6$ µm). D) TLM analysis at the largest gate-overdrive voltage (V_{GS}-V_{th}) before bending, 72 h later (but still before bending), in the bent state, and after bending. E) Contact resistance plotted versus the gate-overdrive voltage.

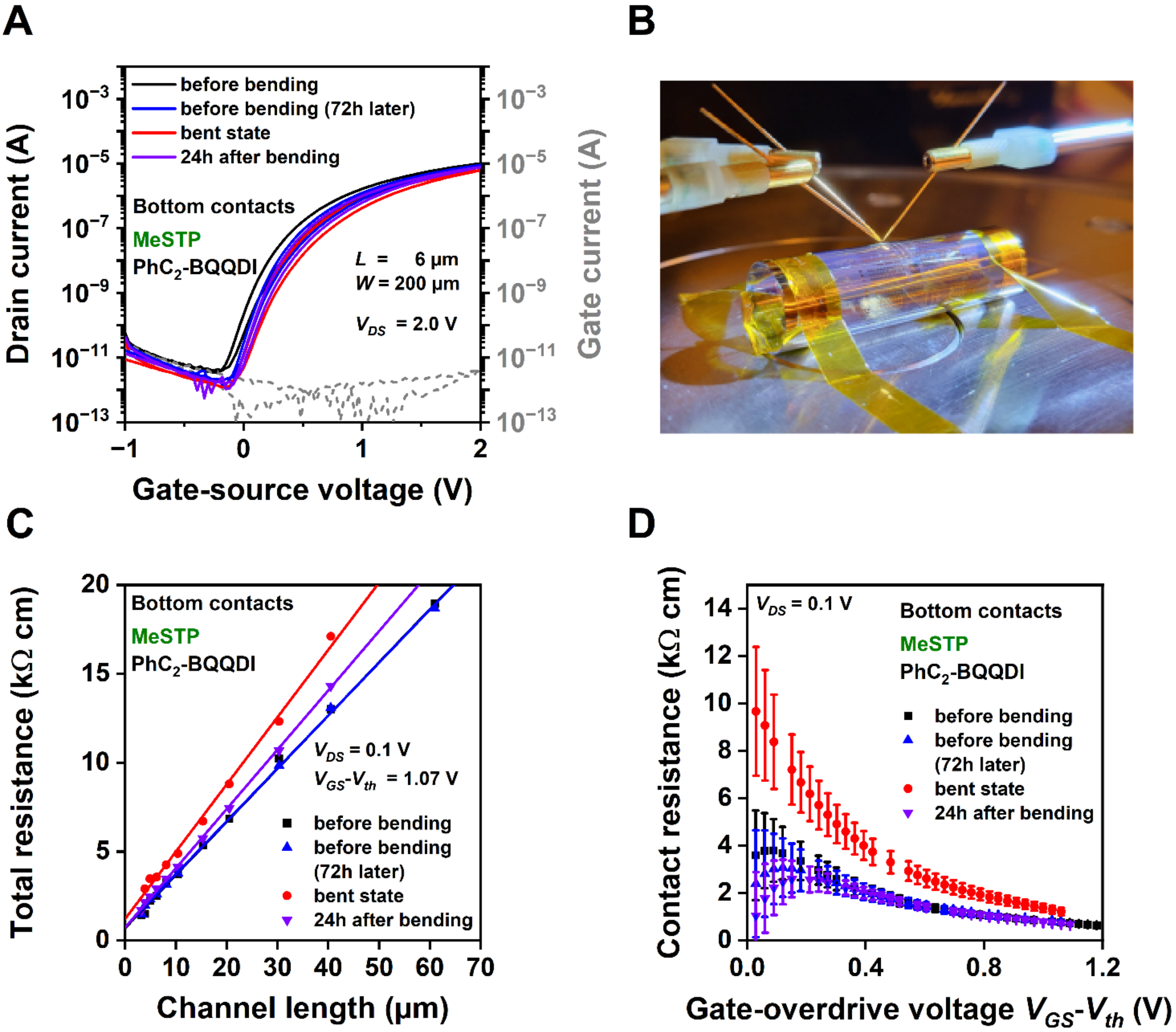

Figure S20. Bending stability of bottom-contact PhC_2-BQQDI TFTs fabricated on a flexible PEN substrate using MeSTP for the contact functionalization. A) Transfer characteristics of a TFT (W = 200 µm, L = 6 µm) before bending, 72 h later (but still before bending), in the bent state, and after bending. B) Photograph of TFTs being measured in the bent state (bending radius ≈ 6 mm). C) TLM analysis at a gate-overdrive voltage (V_{GS}-V_{th}) of 1.07 V of TFTs before bending, 72 h later (but still before bending), in the bent state, and after bending. D) Contact resistance plotted versus the gate-overdrive voltage.

Table S3. Optimized coordinates at B3LYP-GD3(BJ)/Def2-TZVPP level of theory.

xyz-coordinates for TAPP-Br_4:

C	-3.544230000000	-1.204517000000	0.373836000000
C	-0.705814000000	-1.242365000000	0.074235000000
C	-1.389704000000	-0.000254000000	0.147347000000
C	-2.798933000000	-0.000311000000	0.296653000000
C	-3.544318000000	1.203841000000	0.373893000000
C	-0.705908000000	1.241913000000	0.074273000000
C	-1.469048000000	2.445526000000	0.151944000000
N	-4.872260000000	1.188358000000	0.514780000000
C	0.705819000000	1.241969000000	-0.074228000000
C	1.389709000000	-0.000143000000	-0.147344000000
C	0.705915000000	-1.242309000000	-0.074269000000
C	2.798938000000	-0.000087000000	-0.296654000000
C	3.544233000000	1.204123000000	-0.373846000000
C	1.468867000000	2.445642000000	-0.151862000000
C	3.544326000000	-1.204235000000	-0.373885000000
N	4.872181000000	1.188750000000	-0.514719000000
C	5.449587000000	0.000017000000	-0.582128000000
H	-0.969329000000	3.398419000000	0.096386000000
H	0.969075000000	3.398495000000	-0.096278000000
C	-2.820638000000	2.440888000000	0.293662000000
C	2.820457000000	2.441112000000	-0.293576000000
N	4.872276000000	-1.188756000000	-0.514749000000
C	2.820645000000	-2.441282000000	-0.293661000000
C	1.469054000000	-2.445921000000	-0.151946000000
H	0.969335000000	-3.398814000000	-0.096397000000
C	-1.468861000000	-2.446036000000	0.151876000000
C	-2.820454000000	-2.441504000000	0.293583000000
H	-0.969069000000	-3.398890000000	0.096303000000
N	-4.872190000000	-1.189147000000	0.514664000000
C	-5.449588000000	-0.000428000000	0.582105000000
Br	3.772577000000	4.070377000000	-0.387178000000
Br	3.772886000000	-4.070473000000	-0.387326000000
Br	-3.772565000000	-4.070774000000	0.387202000000
Br	-3.772884000000	4.070076000000	0.387318000000
C	6.973500000000	0.000122000000	-0.729104000000
C	7.684334000000	0.000682000000	0.657990000000
C	9.241670000000	0.000066000000	0.633676000000
C	-6.973505000000	-0.000305000000	0.729090000000
C	-7.684353000000	0.003063000000	-0.657983000000
C	-9.241679000000	-0.001098000000	-0.633678000000
F	9.688915000000	0.001157000000	1.894376000000
F	9.712052000000	1.084760000000	0.017268000000
F	-9.688935000000	0.005542000000	-1.894360000000
F	-9.708897000000	-1.091552000000	-0.025128000000
F	-7.293527000000	1.102473000000	-1.337472000000
F	-7.288014000000	-1.089443000000	-1.345619000000
F	7.290335000000	-1.094756000000	1.342185000000
F	7.291161000000	1.097179000000	1.340908000000
F	7.375234000000	-1.094299000000	-1.411789000000
F	7.374975000000	1.094156000000	-1.412557000000
F	-7.374716000000	1.092546000000	1.414612000000
F	-7.375484000000	-1.095909000000	1.409714000000
F	9.711247000000	-1.086211000000	0.019458000000
F	-9.714418000000	1.079371000000	-0.011643000000

xyz-coordinates for N1100:

C	-3.154528000000	-1.059609000000	0.769541000000
C	-0.445921000000	-0.904665000000	-0.014040000000
C	-1.193985000000	0.296961000000	0.177530000000
C	-2.574017000000	0.197621000000	0.510503000000
C	-3.363620000000	1.361747000000	0.595560000000
C	-0.587154000000	1.579099000000	0.051429000000
C	-1.427175000000	2.706743000000	0.074033000000
C	0.868970000000	1.638450000000	-0.077426000000
C	1.577847000000	0.451882000000	-0.437843000000
C	0.923398000000	-0.810055000000	-0.518969000000
C	2.970379000000	0.543517000000	-0.715580000000
C	3.655992000000	1.758990000000	-0.524944000000
C	1.596173000000	2.801693000000	0.145092000000
C	3.668257000000	-0.582836000000	-1.195296000000
H	1.105818000000	3.693899000000	0.490623000000
C	-2.802277000000	2.586566000000	0.344929000000
C	2.972444000000	2.862741000000	-0.067928000000
C	3.001052000000	-1.761517000000	-1.404306000000
C	1.640734000000	-1.887241000000	-1.068759000000
C	-1.047606000000	-2.117120000000	0.300286000000
C	-2.381145000000	-2.195886000000	0.698898000000
H	-0.489597000000	-3.034333000000	0.240941000000
H	3.534191000000	-2.605406000000	-1.816093000000
H	-2.827966000000	-3.150825000000	0.933287000000
H	3.513710000000	3.781015000000	0.106146000000
H	-3.425215000000	3.468277000000	0.367477000000
C	5.114115000000	-0.515372000000	-1.499950000000
N	5.763139000000	0.683830000000	-1.176369000000
C	5.107245000000	1.865300000000	-0.789605000000
O	5.721162000000	-1.440864000000	-1.991623000000
O	5.714941000000	2.906938000000	-0.684950000000
C	1.064344000000	-3.156874000000	-1.375159000000
C	-0.990853000000	4.035047000000	-0.214622000000
N	0.666195000000	-4.197198000000	-1.671246000000
N	-0.720477000000	5.128678000000	-0.458334000000
C	-4.583165000000	-1.178571000000	1.133543000000
C	-4.805033000000	1.289506000000	0.924415000000
N	-5.338279000000	0.005517000000	1.102106000000
O	-5.497182000000	2.276286000000	1.041035000000
O	-5.092369000000	-2.232022000000	1.444492000000
C	8.050503000000	0.528191000000	-0.159951000000
C	8.123044000000	-0.941474000000	0.337865000000
C	9.100142000000	-1.222729000000	1.520167000000
C	-7.572989000000	-0.282827000000	0.117497000000
C	-9.091268000000	-0.389523000000	0.422334000000
C	-10.037948000000	-0.585295000000	-0.798153000000
F	8.502116000000	-1.729870000000	-0.687378000000
F	7.613139000000	1.289825000000	0.871898000000
F	8.992833000000	-2.510909000000	1.862264000000
F	9.316414000000	0.920328000000	-0.466974000000
F	10.362727000000	-0.982693000000	1.167851000000
F	6.888232000000	-1.316514000000	0.750166000000
F	8.796128000000	-0.475983000000	2.583027000000
F	-7.184386000000	-1.406828000000	-0.536451000000
F	-9.476548000000	0.740743000000	1.057759000000
F	-9.283735000000	-1.436348000000	1.257158000000
F	-9.752971000000	-1.714882000000	-1.445996000000
F	-11.297430000000	-0.654142000000	-0.355611000000
F	-7.375799000000	0.758100000000	-0.730812000000
F	-9.940910000000	0.438984000000	-1.645775000000
C	-6.760244000000	-0.096364000000	1.397606000000

H	-7.080596000000	0.818883000000	1.884021000000
H	-6.920438000000	-0.955325000000	2.040577000000
C	7.197149000000	0.762724000000	-1.410571000000
H	7.469863000000	0.035603000000	-2.167878000000
H	7.438301000000	1.768487000000	-1.742741000000

xyz-coordinates for PhC_2-BQQDI:

C	3.526103000000	1.246097000000	-0.145027000000
C	0.709603000000	1.259202000000	-0.027763000000
C	1.409758000000	0.024610000000	-0.059196000000
C	2.821028000000	0.027319000000	-0.118812000000
C	3.497263000000	-1.204154000000	-0.147202000000
C	0.751940000000	-1.234913000000	-0.032856000000
C	-0.709603000000	-1.259198000000	0.027763000000
C	-1.409759000000	-0.024606000000	0.059196000000
C	-0.751941000000	1.234917000000	0.032855000000
C	-2.821028000000	-0.027314000000	0.118813000000
C	-3.526103000000	-1.246092000000	0.145030000000
C	-1.428706000000	-2.443541000000	0.054699000000
C	-3.497263000000	1.204158000000	0.147203000000
H	-0.886034000000	-3.376523000000	0.029995000000
C	2.751681000000	-2.367540000000	-0.115546000000
C	-2.827220000000	-2.435812000000	0.111827000000
C	-2.751681000000	2.367544000000	0.115546000000
C	1.428705000000	2.443545000000	-0.054699000000
C	2.827220000000	2.435816000000	-0.111825000000
H	0.886034000000	3.376527000000	-0.029995000000
H	3.382551000000	3.362639000000	-0.131083000000
H	-3.382551000000	-3.362635000000	0.131086000000
C	-4.971290000000	1.245800000000	0.206668000000
N	-5.627370000000	0.010847000000	0.259763000000
C	-5.004596000000	-1.239934000000	0.204425000000
O	-5.596939000000	2.287831000000	0.211563000000
O	-5.658212000000	-2.264724000000	0.207217000000
C	5.004597000000	1.239939000000	-0.204415000000
C	4.971289000000	-1.245794000000	-0.206672000000
N	5.627370000000	-0.010842000000	-0.259762000000
O	5.596940000000	-2.287826000000	-0.211574000000
O	5.658211000000	2.264730000000	-0.207210000000
C	-7.098580000000	0.030446000000	0.317610000000
C	7.098579000000	-0.030445000000	-0.317613000000
C	-7.722085000000	0.036624000000	-1.071184000000
H	-7.378403000000	0.922584000000	0.869125000000
H	-7.402430000000	-0.851845000000	0.872273000000
H	-7.412141000000	0.919486000000	-1.628981000000
H	-8.809106000000	0.051268000000	-0.988521000000
H	-7.435822000000	-0.855740000000	-1.626485000000
C	7.722087000000	-0.036677000000	1.071180000000
H	7.402430000000	0.851868000000	-0.872241000000
H	7.378400000000	-0.922561000000	-0.869165000000
H	7.435829000000	0.855669000000	1.626513000000
H	8.809108000000	-0.051323000000	0.988514000000
H	7.412140000000	-0.919557000000	1.628946000000
N	-1.412884000000	2.385910000000	0.060751000000
H	-3.255717000000	3.324416000000	0.135800000000
N	1.412884000000	-2.385906000000	-0.060751000000
H	3.255717000000	-3.324412000000	-0.135802000000

Computational methods:

All density-functional-theory (DFT) calculations were performed in the gas phase using the Gaussian 16 program suite (G16RevC.01).[1] The B3LYP functional was used to optimize the geometries on the valence triple-ζ basis set Def2-TZVPP.[2–6] The resulting ground-state structures were confirmed as energy minima through frequency calculations showing no negative eigenvalue in the Hessian matrix. Grimme's dispersion correction D3 with Becke-Johnson damping was considered in all calculations.[7] In the case of PhC_2-BQQDI, phenylethyl substituents were replaced by ethyl groups to reduce computational cost. Table S3 provides a listing of the xyz coordinates of the optimized structures of the calculated semiconductor molecules.

References

(1) Gaussian 16 Revision C.01, M. J. Frisch, G. W. Trucks, H. B. Schlegel, G. E. Scuseria, M. A. Robb, J. R. Cheeseman, G. Scalmani, V. Barone, G. A. Petersson, H. Nakatsuji, X. Li, M. Caricato, A. V. Marenich, J. Bloino, B. G. Janesko, R. Gomperts, B. Mennucci, H. P. Hratchian, J. V. Ortiz, A. F. Izmaylov, J. L. Sonnenberg, D. Williams-Young, F. Ding, F. Lipparini, F. Egidi, J. Goings, B. Peng, A. Petrone, T. Henderson, D. Ranasinghe, V. G. Zakrzewski, J. Gao, N. Rega, G. Zheng, W. Liang, M. Hada, M. Ehara, K. Toyota, R. Fukuda, J. Hasegawa, M. Ishida, T. Nakajima, Y. Honda, O. Kitao, H. Nakai, T. Vreven, K. Throssell, J. A., Jr. Montgomery, J. E. Peralta, F. Ogliaro, M. J. Bearpark, J. J. Heyd, E. N. Brothers, K. N. Kudin, V. N. Staroverov, T. A. Keith, R. Kobayashi, J. Normand, K. Raghavachari, A. P. Rendell, J. C. Burant, S. S. Iyengar, J. Tomasi, M. Cossi, J. M. Millam, M. Klene, C. Adamo, R. Cammi, J. W. Ochterski, R. L. Martin, K. Morokuma, O. Farkas, J. B. Foresman, D. J. Fox, Gaussian, Inc., Wallingford CT, **2016**.

(2) Lee, C.; Yang, W.; Parr, R. G. Development of the Colle-Salvetti Correlation-Energy Formula into a Functional of the Electron Density. *Phys. Rev. B* **1988**, *37* (2), 785–789. https://doi.org/10.1103/PhysRevB.37.785.

(3) Stephen, P. J.; Devlin, F. J.; Chabalowski, C. F.; Frisch, M. J. Ab Initio Calculation of Vibrational Absorption. *J. Phys. Chem.* **1994**, *98* (45), 11623–11627.

(4) Becke, A. D. Thermochemistry. III. The Role of Exact Exchange. *J. Chem. Phys.* **1993**, *98* (December 1992), 5648–5652.

(5) Weigend, F.; Ahlrichs, R. Balanced Basis Sets of Split Valence, Triple Zeta Valence and Quadruple Zeta Valence Quality for H to Rn: Design and Assessment of Accuracy. *Phys. Chem. Chem. Phys.* **2005**, *7* (18), 3297–3305. https://doi.org/10.1039/b508541a.

(6) Weigend, F. Accurate Coulomb-Fitting Basis Sets for H to Rn. *Phys. Chem. Chem. Phys.* **2006**, *8* (9), 1057–1065. https://doi.org/10.1039/b515623h.

(7) Grimme, S.; Ehrlich, S.; Goerigk, L. Effect of the Damping Function in Dispersion Corrected Density Functional Theory. *J. Comput. Chem.* **2011**, *32* (7), 1456–1465. https://doi.org/10.1002/jcc.21759.
